# A Recognition Method of Soybean Leaf Diseases Based on an Improved Deep Learning Model

**DOI:** 10.3389/fpls.2022.878834

**Published:** 2022-05-31

**Authors:** Miao Yu, Xiaodan Ma, Haiou Guan, Meng Liu, Tao Zhang

**Affiliations:** ^1^College of Information and Electrical Engineering, Heilongjiang Bayi Agricultural University, Daqing, China; ^2^College of Information and Electrical Engineering, China Agricultural University, Beijing, China

**Keywords:** soybean diseases, attention mechanism, shortcut connections, residual network, recognition model

## Abstract

Soybean is an important oil crop and plant protein source, and phenotypic traits' detection for soybean diseases, which seriously restrict yield and quality, is of great significance for soybean breeding, cultivation, and fine management. The recognition accuracy of traditional deep learning models is not high, and the chemical analysis operation process of soybean diseases is time-consuming. In addition, artificial observation and experience judgment are easily affected by subjective factors and difficult to guarantee the accuracy of the objective. Thus, a rapid identification method of soybean diseases was proposed based on a new residual attention network (RANet) model. First, soybean brown leaf spot, soybean frogeye leaf spot, and soybean phyllosticta leaf spot were used as research objects, the OTSU algorithm was adopted to remove the background from the original image. Then, the sample dataset of soybean disease images was expanded by image enhancement technology based on a single leaf image of soybean disease. In addition, a residual attention layer (RAL) was constructed using attention mechanisms and shortcut connections, which further embedded into the residual neural network 18 (ResNet18) model. Finally, a new model of RANet for recognition of soybean diseases was established based on attention mechanism and idea of residuals. The result showed that the average recognition accuracy of soybean leaf diseases was 98.49%, and the F1-value was 98.52 with recognition time of 0.0514 s, which realized an accurate, fast, and efficient recognition model for soybean leaf diseases.

## Introduction

Soybean is an important oil crop and plant protein source originating from China (Sun et al., 2021), and phenotypic traits' detection for soybean diseases, which seriously restrict yield and quality (Cen et al., 2020), is of great significance for high-quality breeding, scientific cultivation, and fine management. According to an analysis of relevant investigation data, the yield loss of soybean caused by diseases accounts for about 10% every year and even more than 30% in serious cases (Chang et al., 2018; Guo et al., 2021). Especially in recent years, with the aggravation of environmental pollution, disease stress is more and more intense. Rapid recognition and monitoring of soybean diseases are key and core issues in the morphological-physiological phenotypic detection system of soybean growth process, to achieve not only precise disease control and variable application but also reduction in pesticide residues according to situation and, ultimately, improvement in crop quality and yield. With the rapid development of deep learning in smart agriculture, it has been widely applied in the detection of diseases and pests, recognition of flowers and fruits, classification of plant species, and other fields (Singh et al., 2016). Convolutional neural network (CNN) training models of four deep learning networks (SqueezeNet, ResNet, InceptionV3, and DenseNet) were used to identify four similar rose flowers (Wang et al., 2021). Based on the convolutional neural network of a multi-feature fusion, 32 kinds of leaves in the Flavin database and 189 kinds of leaves in the MEW2014 database were identified and classified, respectively, with average correct recognition rates of 93.25 and 96.37% (Han and Zeng, 2021). Based on the TensorFlow platform, a convolutional neural network model with two convolutional layers was established and trained through MNIST data sets (Liang et al., 2019). Two training methods of TOTV and TVTV convolutional neural networks are proposed and validated with MNIST and CIFAR10 standard image data sets (Kannojia and Jaiswal, 2018). An extended CNN model is constructed and successfully applied for handwritten digit recognition of Mnist data sets (Lei et al., 2019). An 8-layer convolutional neural network was designed to effectively identify plant species with simple and complex backgrounds in the PlantNet leaf library and self-collected leaf images (Zhang and Huai, 2016). A method of grape leaf disease identification based on multi-scale ResNet was proposed (He et al., 2021). Based on the residual network (ResNet18), an improved multi-scale ResNet lightweight disease recognition model is proposed (Wang et al., 2020). A plant species recognition method based on ResNet101 and transfer learning is established and used for an expanded wild plant data set (Li et al., 2021). The weed classification models of ResNet50, VGGNet, and AlexNet networks based on deep learning were built with 10 common weed images in tea gardens as data samples (Gao et al., 2021). Based on a traditional residual error of a neural network, an attention module was adopted to construct a residual network based on a hybrid attention mechanism for recognition of plant diseases through Plant Village public data sets of apple and cherry, and a total of 10 kinds of crops, such as corn, grapes, and citrus, and 60 kinds of diseases were tested; the recognition accuracy reached 92.08% (Shang et al., 2021). Based on ResNet50, a parallel pooled attention module was constructed, and a residual attention network model was proposed to identify four potato diseases in the Plant Village Datase dataset with an accuracy of 93.86% (Xu et al., 2021). An attentional module was embedded into the ResNeXt50 structure of a residual network, and a tomato leaf disease recognition method was proposed based on a 3-channel attentional mechanism network (Ma et al., 2021).

At present, there are few reports on recognition of soybean leaf diseases based on deep learning models. However, traditional disease diagnosis methods based on image processing have a complex feature extraction process and poor adaptive ability. The chemical analysis operation process is complicated and time-consuming. Artificial visual observation and empirical judgment are easily affected by subjective factors, and it is difficult to ensure objectivity and accuracy. Therefore, a novel deep convolutional neural network based on attention mechanism and residual idea is proposed in this study, and a novel residual attention network model (RANet) for soybean disease recognition is established by identity mapping with a shortcut connection. First, three leaf diseases, namely, soybean brown leaf spot, soybean frogeye leaf spot, and soybean phyllosticta leaf spot, were used as research objects, the background of an original image was removed with the OTSU algorithm, and a single leaf image of soybean disease was obtained with the region labeling method. Then, the image data of single leaf disease were enhanced by rotation, mirroring, noise addition, and filtering to expand soybean disease image samples. In addition, two continuous convolution layers of ResNet18 were used to construct a residual attention layer (RAL) by adding attention mechanism and shortcut connection. The RAL was embedded in ResNet18 to replace the residual structure. Finally, a new residual attention network (RANet) was established to realize fast recognition of soybean leaf diseases, which could provide theoretical basis and technical support for intelligent mining and analysis of phenotype big data for crop diseases.

## Materials and Methods

### Overall Process of Establishing the Recognition Model

**Figure 1:**
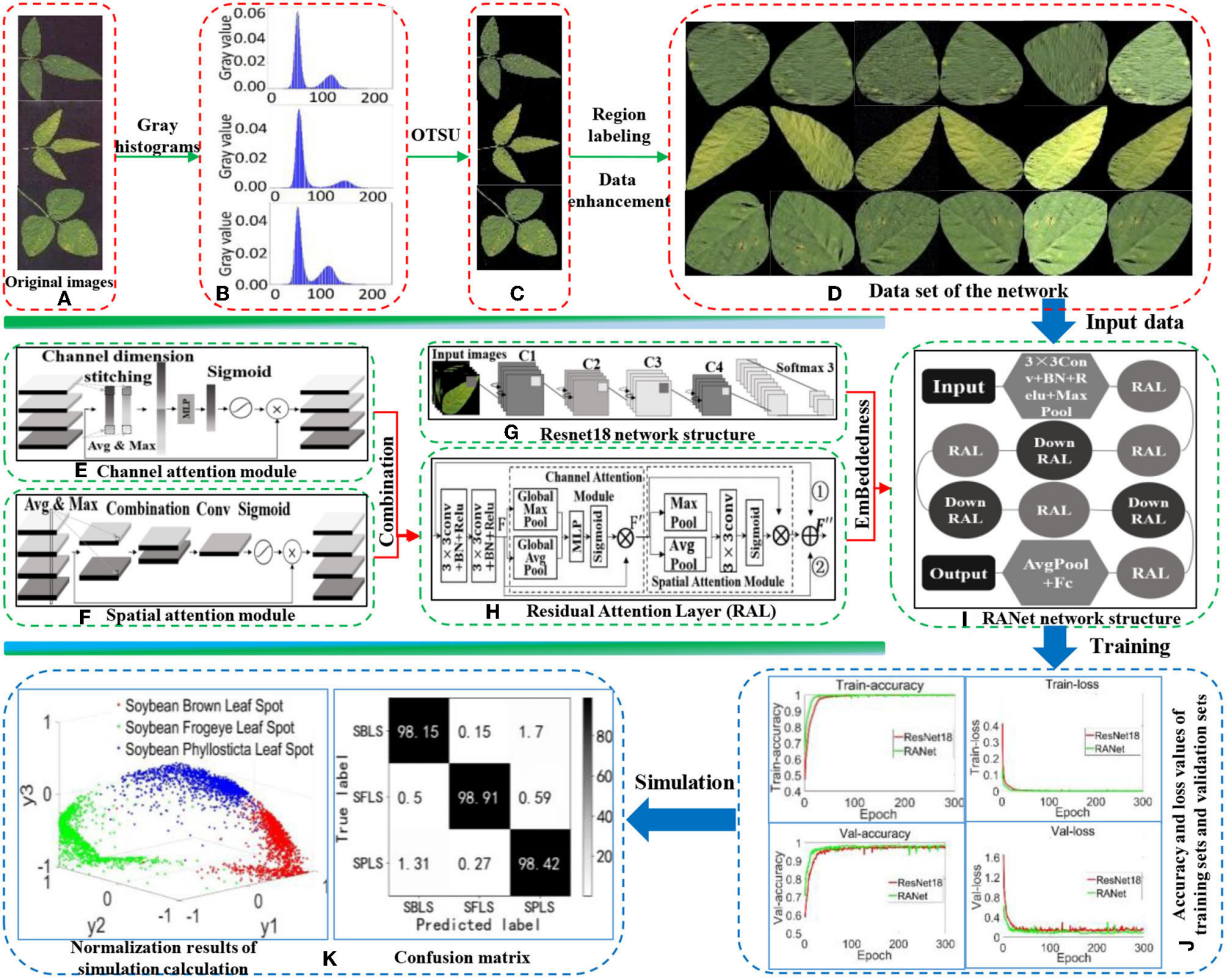
Overall process of establishing the recognition model. **(A)** Original images. **(B)** Gray histogram. **(C)** The extracted images by OTSU. **(D)** Data set of the network. **(E)** Channel attention module. **(F)** Spatial attention module. **(G)** Resnet18 network structure. **(H)** Residual Attention Layer (RAL). **(I)** RANet network structure. **(J)** Accuracy and loss values of training sets and validation sets. **(K)** Normalization results of simulation calculation, confusion matrix.

The overall process of establishing the recognition model for soybean leaf diseases is shown in Figure 1. First, the background of soybean brown leaf spot, soybean frogeye leaf spot, and soybean phyllosticta leaf spot was removed with the OTSU algorithm. Second, the region calibration method was used to obtain the image of soybean disease from a single leaf. Then, the image data of single leaf disease were enhanced by rotation, mirroring, noise addition, and filtering for expanding the image samples of soybean leaf diseases. In addition, the RAL was constructed by combining the attention mechanism with shortcut connection based on two continuous convolution layers of ResNet18, and was embedded into ResNet18 to replace the residual structure. Finally, a new RANet was established to realize fast recognition of soybean leaf diseases. Accuracy rate, recognition rate, F1-value, and recognition time were used to evaluate the validity of the recognition model.

### Experiment Materials

**Figure 2:**
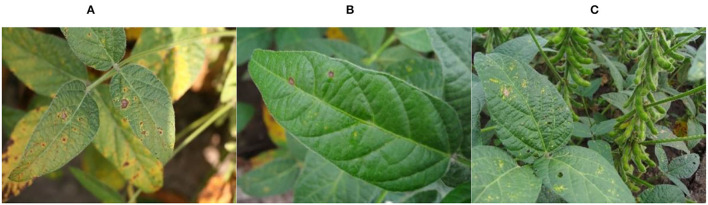
Images of the soybean diseases. **(A)** Soybean brown leaf spot; **(B)** soybean frogeye leaf spot; **(C)** soybean phyllosticta leaf spot.

The data samples of this study were acquired by plant protection experts from Heilongjiang Bayi Agricultural University from 2006 to 2021 on soybean production experiment fields of 850 farms in Heilongjiang province, China. Soybean brown leaf spot is caused by fungi of Ascomycota, Myriangiales, and Mycosphaerella sojae Hori of Mycosphaerella. The Soybean brown leaf spot is irregularly shaped with 2–5 mm in diameter, it is light brown or grayish brown with dark brown edge, which has obvious boundary compared to the healthy part. Disease spots are dry, light color and grayish white, and there are obvious small black spots on disease spots. Soybean Frogeye Leaf Spot is caused by fungi of Deuteromycota, Hyphomycetales, Dermataceae, and Cercosporidium sojinum (Hara) Liu and Guo of Cercosporidium. After infection, the leaves of an adult plant show a round, oval, or irregular shape, gray in the center and brown in the edge, with an obvious boundary between the diseased spot and healthy tissue, resembling frog's eye. When wet, there is dense gray mildew on the back of the diseased spot, which is the conidia of the pathogen. In severe cases, the leaves are full of disease bands that can merge with each other and make the leaves dry up. The soybean phyllosticta leaf spot is caused by fungi of Deuteromycota, Sphacropsidales, and Phyllosticta sojaecola Massal of Phyllosticta. Leaf lesions are round, oval or irregular in shape with a diameter of 2–5 mm, light brown at the beginning, with a very fine dark brown edge, and grayish white at the later stage. Soybean varieties Kenong 23, Kenfeng 17, Heonong 44, Xiannong 1, Dongnongdou 252, and Kendou 94 were mainly planted in the second accumulation zone in this test area. Images of diseased soybean leaves were collected from soybean plants of the above varieties. The image data included 523 images of soybean brown leaf spot, soybean frogeye leaf spot, and soybean phyllosticta leaf spot in the full flowering stage (R2), beginning pod stage (R3), full pod stage (R4), beginning seed stage (R5), and full seed stage (R6). The three kinds of diseases are shown in Figure 2.

### Image Segmentation

In this study, an iterative method, OTSU, and a local threshold method were used to extract the diseased spots from the background of original soybean leaf images.

The threshold segmentation method was selected due to the significant difference between the gray level of soybean diseased leaves and the shooting background after the original image of soybean diseased leaves was transformed into a gray level image. The image thresholding segmentation is suitable for images with different gray level ranges of target and background. The selection of threshold value in the method will directly affect the segmentation effect of soybean diseased leaves. An original image of soybean diseased leaves was set as *f*(*x, y*), and the image after segmentation with single threshold value T was defined as:


(1) $$\begin{array}{l} g\left(x,y\right)=\{\begin{array}{cc} 0 & f\left(x,y\right)<T\\ 1 & f\left(x,y\right)>T \end{array} \end{array}$$

where *g*(*x, y*) represented the binary image.

After segmentation, the binary image *g*(*x, y*) divided the original soybean disease leaf image *f*(*x, y*) into two parts: soybean diseased leaves and the background.

(1) Iterative method

Based on the idea of approximation, the iterative method can automatically select the threshold value according to image data. Assume that the image has a total of L gray scale, the gray median value of soybean diseased images was taken first as the initial threshold of segmentation.


(2) $$\begin{array}{l} T_{i+1}\;=\;\frac{1}{2}\left\{\frac{\sum_{k\;=\;0}^{T_{i}}h_{k}\cdot k}{\sum_{k\;=\;0}^{T_{i}}h_{k}}+\frac{\sum_{k\;=\;T_{i}\;+\;1}^{L\;-\;1}h_{k}\cdot k}{\sum_{k\;=\;T_{i}\;+\;1}^{L-1}h_{k}}\right\} \end{array}$$

Where *h*_*k*_ was the number of pixels with a gray value of K in the gray image of soybean diseases. When *T*_*i*+1_ = *T*_*i*_, the iteration ended, and *T*_*i*_ was the final segmentation threshold.

The specific steps were as follows:

① The maximum gray value and minimum gray value of the original image *f*(*x, y*) of soybean diseases were calculated and denoted as *Z*_max_ and *Z*_min_, respectively, and the initial threshold t was calculated as follows:


(3) $$\begin{array}{l} T_{0}\;=\;\frac{1}{2}\left(Z_{\max}\;+\;Z_{\min}\right) \end{array}$$

② According to the threshold value *T*_*k*_, the soybean image was divided into leaf and background, and the average gray values *Z*_*a*_ and *Z*_*b*_ were calculated.

③ A new threshold of soybean disease image segmentation was obtained:


(4) $$\begin{array}{l} T_{k}\;=\;\frac{1}{2}\left(Z_{a}\;+\;Z_{b}\right) \end{array}$$

④ If *T*_*k*_ = *T*_*k* + 1_, the separation effect of diseased leaves and background in soybean image, was the best, and the obtained value was the threshold value, otherwise, go to ② to continue to calculate the average gray value of soybean diseased leaves and background, and do iterative calculation.

⑤ According to the obtained threshold *T*_*k*_, the soybean disease image was binarized and segmented. The segmentation equation was as follows:


(5) $$\begin{array}{l} g_{1}\left(x,y\right)\;=\;\{\begin{array}{cc} 0 & f\left(x,y\right)\;<\;T_{k}\\ 1 & f\left(x,y\right)\;>\;T_{k} \end{array} \end{array}$$

⑥ Restore the RGB value of the original soybean disease image *f*(*x, y*) to the diseased soybean leaf in the segmented binary image *g*_1_(*x, y*).

**Figure 3:**
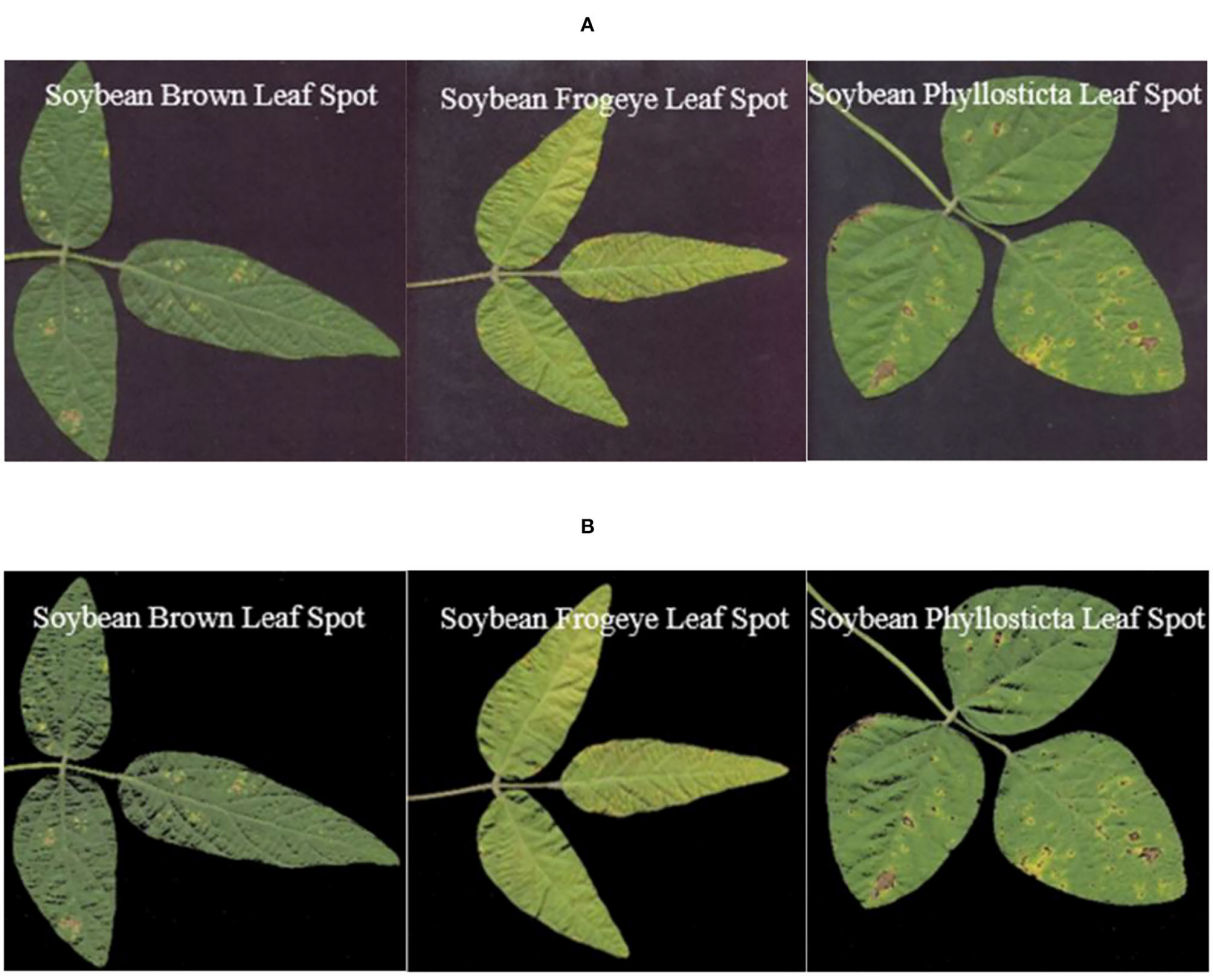
Segmented images of soybean diseased leaves with the iterative method. **(A)** Original images. **(B)** Segmented images.

The effects of the iterative method before and after the segmentation of images of three kinds of soybean diseased leaves are shown in Figure 3.

(2) OTSU algorithm

The OTSU algorithm is an adaptive threshold determination method. The idea was to divide the soybean gray histogram into two groups at a certain threshold value. When the variance of the two groups is the maximum, the threshold value is obtained. Because variance is a measure of the uniformity of gray distribution, the larger the variance value, the greater the difference between diseased leaves and background of soybean image. When some leaves are misclassified into background or some background is misclassified into leaves, the difference between the two parts becomes smaller. Therefore, the segmentation with the largest inter-class variance means the lowest misclassification probability.

It was assumed that there was a threshold *T*_*h*_, and we divided all the pixels of soybean disease image *f*(*x, y*) into two categories: soybean disease leaf *C*_1_ (less than *T*_*h*_) and background *C*_2_ (greater than *T*_*h*_), then the mean values of these two categories of pixels were *m*_1_ and *m*_2_, respectively, and the mean values of global pixels of the image were *m*_*G*_, the probability of the pixels being classified into *C*_1_ and *C*_2_ was *p*_1_ and *p*_2_, respectively. The specific steps were as follows:

① To calculate the global pixel mean of the image *m*_*G*_, the calculation formula was:


(6) $$\begin{array}{l} m_{G}\;=\;p_{1}\times m_{1}\;+\;p_{2}\;\times \;m_{2} \end{array}$$

where *p*_1_ + *p*_2_ = 1. ② According to the concept of variance, the calculation formula of inter-class variance was:


(7) $$\begin{array}{l} \sigma^{2}\;=\;p_{1}{\left(m_{1}\;-\;m_{G}\right)}^{2}\;+\;p_{2}{\left(m_{2}\;-\;m_{G}\right)}^{2} \end{array}$$

We substituted Equation (6) into Equation (7), and the following simplification was obtained:


(8) $$\begin{array}{l} \sigma^{2}\;=\;p_{1}p_{2}{\left(m_{1}\;-\;m_{2}\right)}^{2} \end{array}$$

where


(9) $$\begin{array}{l} p_{1}\;=\;\overset{k}{\underset{i\;=\;0}{\sum }}p_{i}\;k\in \left[0,255\right] \end{array}$$

(10) $$\begin{array}{l} m_{1}\;=\;\frac{1}{p_{1}\;\times \;\overset{k}{\underset{i\;=\;0}{\sum }}ip_{i}}\;k\in \left[0,255\right] \end{array}$$

(11) $$\begin{array}{l} m_{2}=\frac{1}{p_{2}\times \overset{k}{\underset{i=0}{\sum }}ip_{i}}\;k\in \left[0,255\right] \end{array}$$

③ 0 Gray levels, ~255, were traversed. When σ^2^ in the gray level was the largest in Formula (8), the difference between the diseased leaves and the background of the soybean image was the largest. Gray level was the best threshold of the segmentation of the diseased leaves and the background of the soybean image, that is, the desired OTSU threshold *T*_*h*_.

④ According to the obtained threshold *T*_*h*_, a binary segmentation of soybean disease images was performed, and the segmentation formula was as follows:


(12) $$\begin{array}{l} g_{2}\left(x,y\right)=\{\begin{array}{cc} 0 & f\left(x,y\right)<T_{h}\\ 1 & f\left(x,y\right)>T_{h} \end{array} \end{array}$$

⑤ The RGB value of the original soybean disease image *f*(*x, y*) was restored to the diseased soybean leaves in the segmented binarized image *g*_2_(*x, y*).

**Figure 4:**
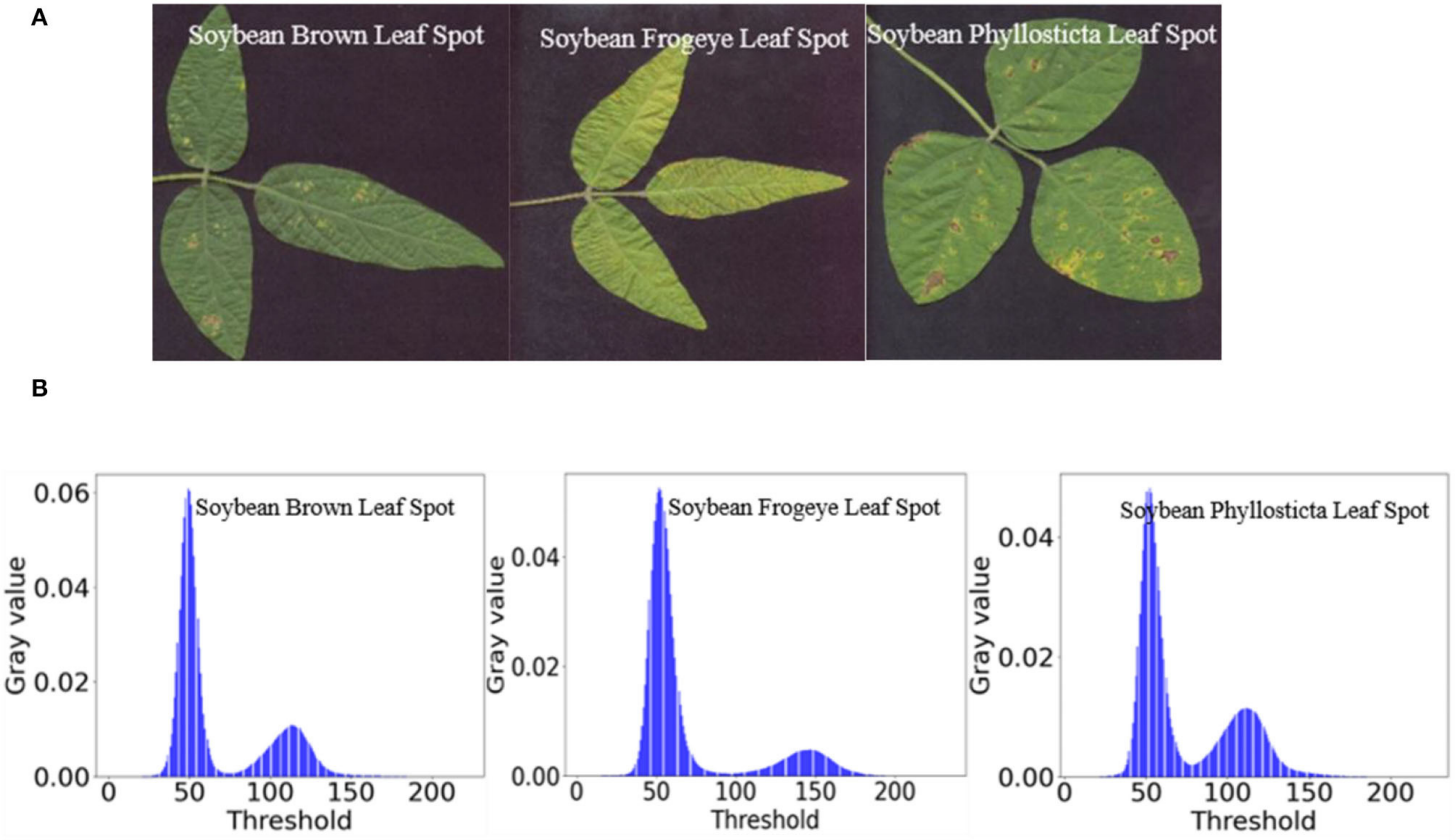
Histogram analysis of soybean disease images. **(A)** Original images. **(B)** Gray histograms.

The image and gray histogram of soybean diseased leaves in the experiment are shown in Figure 4.

Figure 4B shows that the gray histogram of the soybean disease image had obvious double peaks. The threshold values of *T*_1_ = 0.3176, *T*_2_ = 0.3882, and *T*_3_ = 0.3255 after transformation between abscissa [0, 255] are *t*_1_ = 80.9880, *t*_2_ = 98.9910, and *t*_3_ = 83.0025, respectively, and are the locations of the valleys between the three gray histograms and the two peaks, which indicates that the sample data of this experiment are in accordance with the characteristics of the OTSU algorithm.

**Figure 5:**
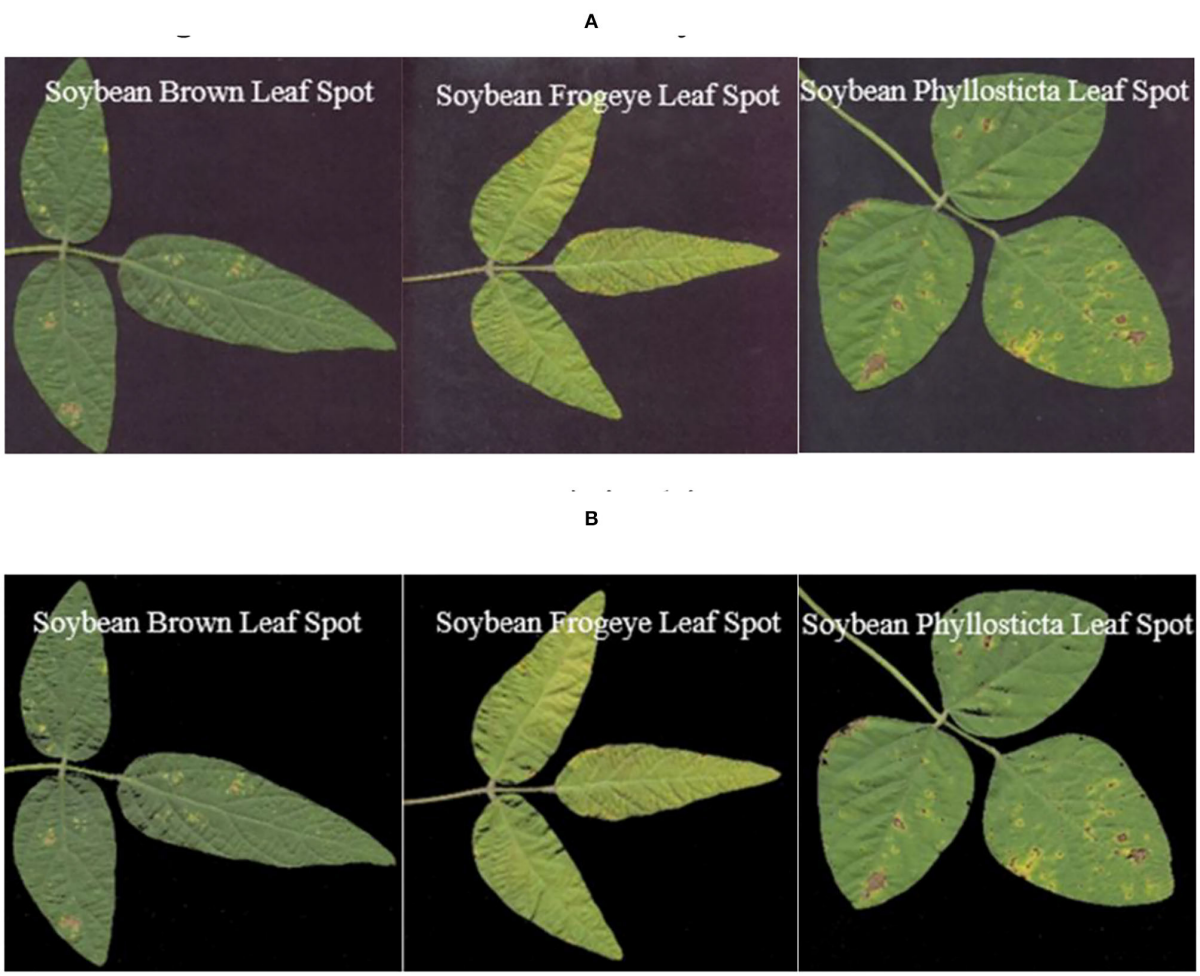
Segmentation of soybean diseased leaves' images with the OTSU algorithm. **(A)** Original images. **(B)** Segmented images.

Figure 5 shows the effects of the OTSU algorithm before and after the segmentation of images of the three kinds of soybean diseased leaves.

(3) Local threshold segmentation method

The local threshold segmentation method uses the threshold function *T*(*x, y*) of local variation to segment soybean disease image *f*(*x, y*), and the specific steps were as follows:

① We created a disk-shaped single structural element SE with a radius of 1,000, that is, all the pixels of the structural element were composed of pixels with a distance ≤ 1,000 from the center pixel.

② We performed top hat transformation. The single structural element SE in ① was used to perform the open operation *f*_0_(*x, y*) on the gray scale image of the input soybean disease image *f*(*x, y*), and then the global threshold segmentation was applied to *f*_0_(*x, y*) to obtain the threshold *T*_0_. Then, the equation of *T*(*x, y*) of the local change threshold function was as follows:


(13) $$\begin{array}{l} T\left(x,y\right)\;=\;f_{0}\left(x,y\right)\;+\;T_{0} \end{array}$$

③ We performed a global threshold segmentation on the basis of the image after open operation, and the segmentation formula was as follows:


(14) $$\begin{array}{l} g_{3}\left(x,y\right)\;=\;\{\begin{array}{cc} 1 & if\;f\left(x,y\right)\geq T\left(x,y\right)\\ 0 & if\;f\left(x,y\right)\;<\;T\left(x,y\right) \end{array} \end{array}$$

④ The RGB value of the original soybean disease image *f*(*x, y*) was restored to the diseased soybean leaf in the segmented binary image *g*_3_(*x, y*).

**Figure 6:**
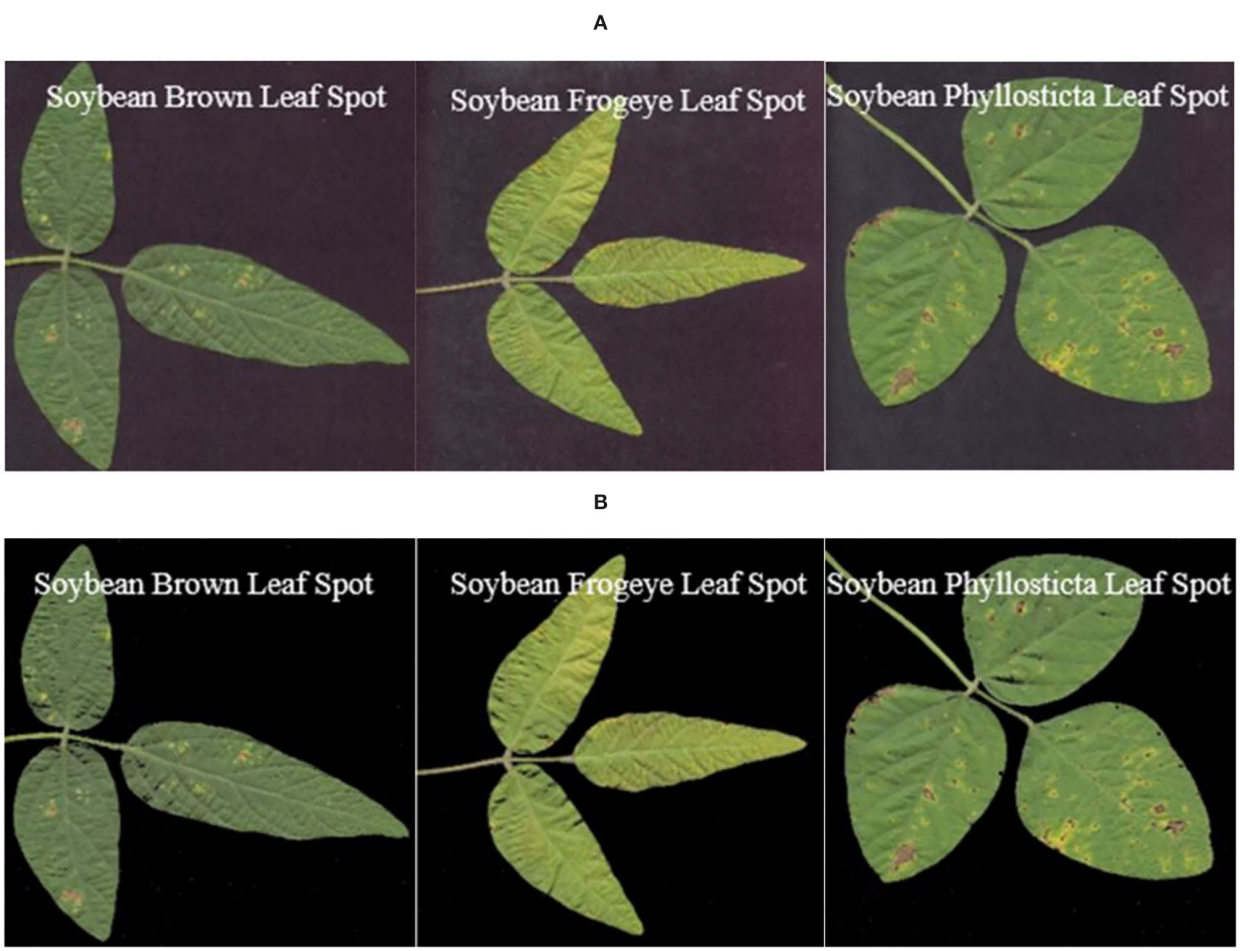
Segmentation of soybean diseased leaves' images with the local threshold segmentation method. **(A)** Original images. **(B)** Segmented images.

The effects of the local threshold segmentation method before and after the segmentation of images of three soybean diseased leaves are shown in Figure 6.

The soybean disease images obtained with the above three segmentation methods could not be visually judged by naked eyes. By analyzing these three segmentation methods and conducting a quantitative analysis, the best segmentation algorithm suitable for the experimental data samples was selected here.

(4) Evaluation index of segmentation quality of target images

① Precision represents the proportion of positive samples (TP + FP) in all positive samples (TP + FP) predicted. The pixels of the real diseased soybean leaf in the image before segmentation were predicted to be the number of pixels of the diseased soybean leaf (TP) in the image after segmentation, and the proportion of the pixels of the real diseased soybean leaf (TP) and the background (FP) in the image before segmentation was predicted to be the number of pixels of the diseased soybean leaf (TP) in the image after segmentation. The definition was as follows:


(15) $$\begin{array}{l} Precision\;=\;\frac{TP}{TP\;+\;FP} \end{array}$$

where true positive (TP) referred to the number of correctly classified positive samples, that is, the predicted positive samples were actually positive samples. In the soybean disease images, TP referred to the number of pixels of the actual soybean diseased leaves that were correctly predicted as the number of pixels of the soybean diseased leaves in the image after segmentation. False positive (FP) referred to the number of negative samples incorrectly marked as positive samples, that is, the actual negative samples were predicted as positive samples. In soybean disease images, FP referred to the number of pixels of the actual image background incorrectly predicted as the pixels of soybean disease leaves. True negative (TN) referred to the number of correctly classified negative samples, that is, the predicted negative samples were actually negative samples. In the soybean disease image, TN referred to the number of background pixels of the actual images that were correctly predicted as image background pixels. False negative (FN) referred to the number of positive samples incorrectly marked as negative samples, that is, the actual positive samples were predicted to be negative samples. In the soybean disease image, FN referred to the number of pixels of the actual soybean diseased leaves incorrectly predicted as image background pixels.

② DICE Coefficient (DICE) is a statistic used to evaluate the similarity of two samples, indicating the ratio of the area intersecting soybean disease images before and after segmentation to the total area. The definition was as follows:


(16) $$\begin{array}{l} DSC\;=\;\frac{2|X\cap Y|}{\left|X|+|Y\right|} \end{array}$$

where |*X*| and |*Y*|, respectively, represented the number of elements in the image before and after segmentation, and the coefficient in the numerator was 2, indicating that there was a common element between |*X*| and |*Y*| in the denominator.

③ Overlap Error (OE) is used to evaluate the overlap error of two samples, and represents the ratio of the overlap error area to the total area of soybean disease images before and after segmentation. The definition was as follows:


(17) $$\begin{array}{l} OE\;=\;\frac{2|X-Y|}{\left|X|+|Y\right|} \end{array}$$

④ Over-segmentation rate (TPR), which means the predicted positive sample is actually a positive sample (TP), accounting for the proportion of all positive samples (TP + FP). The pixels of the real diseased soybean leaves in the images before segmentation were predicted to be the number of diseased soybean leaf pixels (TP) in the images after segmentation. The proportion of the pixels of diseased soybean leaf pixels in the image before segmentation was predicted to be the number of diseased soybean leaf pixels (TP) and background (FP) in the image after segmentation. The definition was as follows:


(18) $$\begin{array}{l} TPR\;=\;\frac{TP}{TP\;+\;FN} \end{array}$$

⑤ Under-segmentation rate (FPR), which represents the ratio of predicted positive samples to all actual negative samples (FP + TN). The pixels of the real background in the image before segmentation were predicted to be the number of pixels (FP) of soybean diseased leaves in the image after segmentation. The proportion of the pixels of the real background in the image before segmentation was predicted to be the number of pixels (FP) and background (TN) of soybean diseased leaves in the image after segmentation. The definition was as follows:


(19) $$\begin{array}{l} FPR\;=\;\frac{FP}{FP\;+\;TN} \end{array}$$

The evaluation index values of the three segmentation methods are shown in Table 1.

**Table 1:** Evaluation of segmentation methods.

** 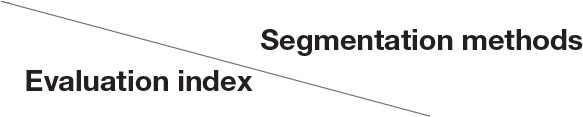 **	**Iterative method**	**OTSU method**	**Local threshold segmentation method**
Precision	1.0000	0.9979	0.2997
DSC	0.9103	0.9989	0.9951
OE	0.1975	0.0021	0.0041
TPR	0.8203	1.0000	0.9931
FPR	0.0000	0.0006	0.0009

The values of five indicators obtained by the three segmentation methods were compared and analyzed on the sample data set of this experiment.

① In the evaluation index precision, the value of perfect segmentation was 1. The iterative method and the OTSU algorithm performed well. The segmentation accuracy of soybean disease images was close to 1, while the local threshold segmentation method performed poorly.

② The range of DSC was (0, 1). In terms of the ratio of intersection area to total area of soybean disease images before and after segmentation, the value of perfect segmentation was 1. The method of OTSU was the best, followed by the local threshold segmentation method.

③ In terms of the ratio of the overlap error area to the total area of soybean disease images before and after segmentation, the value of perfect segmentation was 0, and the method of maximum classes square error was the best, followed by the local threshold segmentation method.

④ In the evaluation index TPR, the value of perfect segmentation was 1, and the method of the OTSU method was the best, followed by the local threshold segmentation method.

⑤ In the evaluation index FPR, the value of perfect segmentation was 0. The iterative method and the OTSU method were the best, followed by local threshold segmentation method.

The comprehensive analysis showed that: compared with the comparative iteration method and the local threshold segmentation method, the OTSU method has the best segmentation effect in the segmentation experiment on data set samples.

### Region Labeling of Diseased Leaves

**Figure 7:**
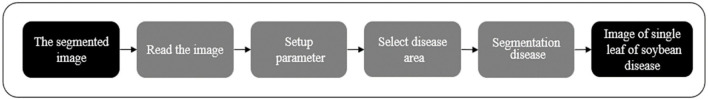
Flow chart of region labeling.

The region labeling was based on the soybean disease images segmented with the OTSU method, and a single leaf with soybean disease was obtained by mouse point selection and image clipping. The flow chart of an image for regional labeling is shown in Figure 7.

The specific steps were as follows:

1. Read an image of soybean disease in the folder after the background segmentation using the method of maximum classes square error.
2. Set the number of points taken by the mouse to 2 in the image (1), and the number of soybean diseased leaves required to be calibrated in the image m. According to the actual number of soybean diseased leaves in the actual shooting data, m was one of the values from 1 to 6.
3. According to the number 2 set in step (2), based on the image coordinate system from left to right X axis and from top to bottom Y axis, selecte the starting point (*x*_1_, *y*_1_) and end point (*x*_2_, *y*_2_) of the rectangular area where the soybean leaf was located with the mouse, calibrated and cut out a soybean leaf for each selected time, and selected M times in total. When clipping an image, (*x*_1_, *y*_1_) represented the position of the top left pixel in the original image after clipping; |*x*_1_ − *x*_2_| represented the height of the image after clipping, and |*y*_1_ − *y*_2_| represented the width of the image after clipping.
4. Save the images of M single leaves with soybean disease selected in step (3) successively.

**Figure 8:**
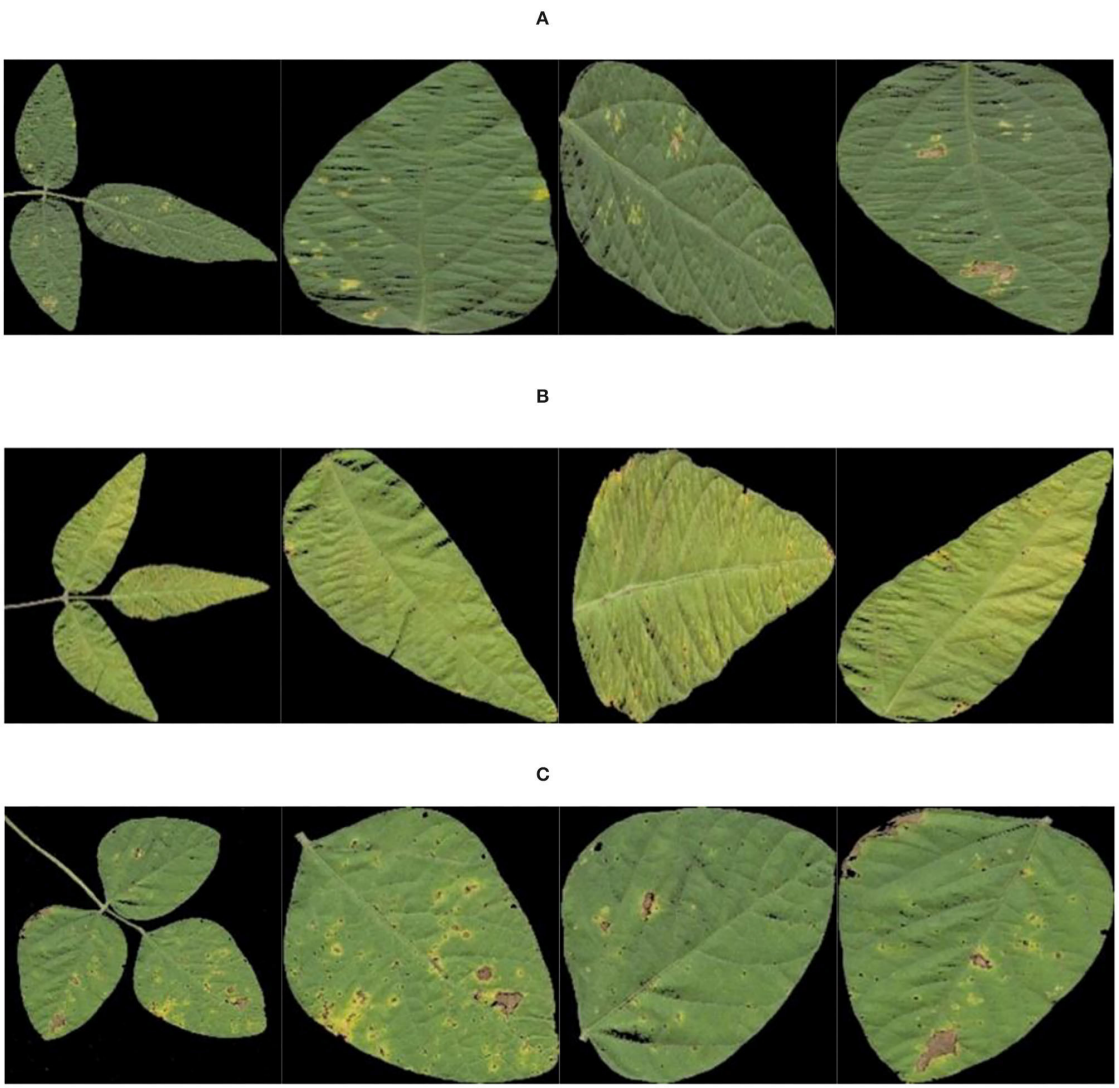
Results of region labeling. **(A)** Soybean brown leaf spot; **(B)** soybean frogeye leaf spot; **(C)** soybean phyllosticta leaf spot.

Figure 8 shows the images of single leaves with soybean disease after segmentation with the OTSU method and images of single leaves with soybean disease obtained with the region labeling method.

### Sample Expansion and Division

Data enhancement took the single leaf image of soybean disease segmented by region labeling as the object. The size of the image was first scaled to 100 × 100, and then the data set images were expanded by Gaussian filtering, image brightening and darkening, reversal, Gaussian noise addition, rotation, and image rotation. The enhancement technology provided a data basis for training model and balance of sample size to improve the generalization ability of the model.

(1) Image rotation: we took the center of the image as the origin and rotated it to a certain angle. The original coordinate of the image pixel was (*x*_0_, *y*_0_), and we obtained (*x*_1_, *y*_1_) after selecting the angle (clockwise). The mathematical formula was expressed as follows:

(20) $$\begin{array}{l} x_{1}\;=\;x_{0}\;\cdot \text{ cos }\theta \;+\;y_{0}\;\cdot \text{sin }\theta \end{array}$$

(21) $$\begin{array}{l} y_{1}\;=\;x_{0}\;\cdot \text{sin }\theta \;+\;y_{0}\;\cdot \text{cos }\theta \end{array}$$

The matrix was shown as follows:


(22) $$\begin{array}{l} \left[\begin{array}{c} x_{1}\\ y_{1}\\ 1 \end{array}\right]\;=\;\left[\begin{array}{ccc} \text{cos }\theta & \text{sin }\theta & 0\\ -\text{sin }\theta & \text{cos }\theta & 0\\ 0 & 0 & 1 \end{array}\right]\left[\begin{array}{c} x_{0}\\ y_{0}\\ 1 \end{array}\right] \end{array}$$

In the single leaf image of soybean disease, the value of θ was 90° and 180°, that is, with the center of the image as the origin, the single leaf image of soybean disease was rotated clockwise by 90° and 180° to obtain the rotated single leaf image of soybean disease.

(2) Horizontal mirror transformation: with the vertical axis of the image as the center, the image was divided into left and right parts for symmetric transformation. The height of the image, H, was associated with the x axis, and the width of the image, W, was associated with the y axis. The coordinate of pixel (*x*_0_, *y*_0_) in the original image became (*x*_0_, *W* − *y*_0_) after horizontal mirror imaging. The equation was expressed as follows:

(23) $$\begin{array}{l} x_{1}\;=\;x_{0},\;y_{1}\;=\;W\;-\;y_{0} \end{array}$$

The matrix was expressed as:


(24) $$\begin{array}{l} \left[\begin{array}{c} x_{1}\\ y_{1}\\ 1 \end{array}\right]\;=\;\left[\begin{array}{ccc} 1 & 0 & 0\\ 0 & -1 & W\\ 0 & 0 & 1 \end{array}\right]\left[\begin{array}{c} x_{0}\\ y_{0}\\ 1 \end{array}\right] \end{array}$$

where $\left[\begin{array}{ccc} 1 & 0 & 0\\ 0 & -1 & W\\ 0 & 0 & 1 \end{array}\right]$ was the horizontal mirror transformation matrix (factor).

In the single leaf image of soybean disease, the image height *H* = 100, the image width *W* = 100, and the single leaf image of soybean disease after horizontal mirror image transformation can be obtained according to Equations (23) and (24).

(3) Gaussian filtering: it is the process of weighted average of the whole image. The value of each pixel is weighted by its own value and the value of other pixels in the neighborhood. The specific steps of Gaussian filtering for the soybean disease single leaf image were as follows:

① In this experiment, a 3 × 3 Gaussian kernel was used for sampling with the central position as the origin of coordinates. The coordinates of each position of the Gaussian kernel were shown in Equation (25) (X-axis was horizontally to the right, and Y-axis was vertically upward). The 3 × 3 Gaussian kernel was shown as follows:


(25) $$\begin{array}{l} G\;=\;\left[\begin{array}{ccc} \left(-1,1\right) & \left(0,1\right) & \left(1,1\right)\\ \left(-1,0\right) & \left(0,0\right) & \left(1,0\right)\\ \left(-1,-1\right) & \left(0,-1\right) & \left(1,-1\right) \end{array}\right] \end{array}$$

② Put the coordinates (*x, y*) of each position of the Gaussian filter in step ① (filtering radius a = 1.5) into the Gaussian function Equation (26), and arrange each value according to the corresponding position. The weight matrix was obtained as shown in Equation (27).

The formula of two-dimensional Gaussian function is:


(26) $$\begin{array}{l} G\left(x,y\right)\;=\;\frac{1}{2\pi \sigma^{2}}e^{-\left(x^{2}+y^{2}\right)/2\sigma^{2}} \end{array}$$

(27) $$\begin{array}{l} W\;=\;\left[\begin{array}{ccc} 0.0453542 & 0.0566406 & 0.0453542\\ 0.0566406 & 0.0707355 & 0.0566406\\ 0.0453542 & 0.0566406 & 0.0453542 \end{array}\right] \end{array}$$

③ The normalization of the weight matrix in step ② aims to make the total weight of the final image channel be 1. The reason is that the weight matrix with a total weight >1 will make the image brighter, while the weight matrix with a total weight <1 will make the image darker. The sum of the 9 weight values is equal to 0.4787147, and the above values are divided by the sum to obtain the normalized weight matrix as shown in Equation (28), which is the final Gaussian filter matrix.


(28) $$\begin{array}{l} W\;=\;\left[\begin{array}{ccc} 0.0947416 & 0.1183180 & 0.0947416\\ 0.1183180 & 0.1477610 & 0.1183180\\ 0.0947416 & 0.1183180 & 0.0947416 \end{array}\right] \end{array}$$

④ The Gaussian filter matrix obtained by step ③ was convolved with the pixel matrix of the image to obtain the image after Gaussian filtering.

**Figure 9:**
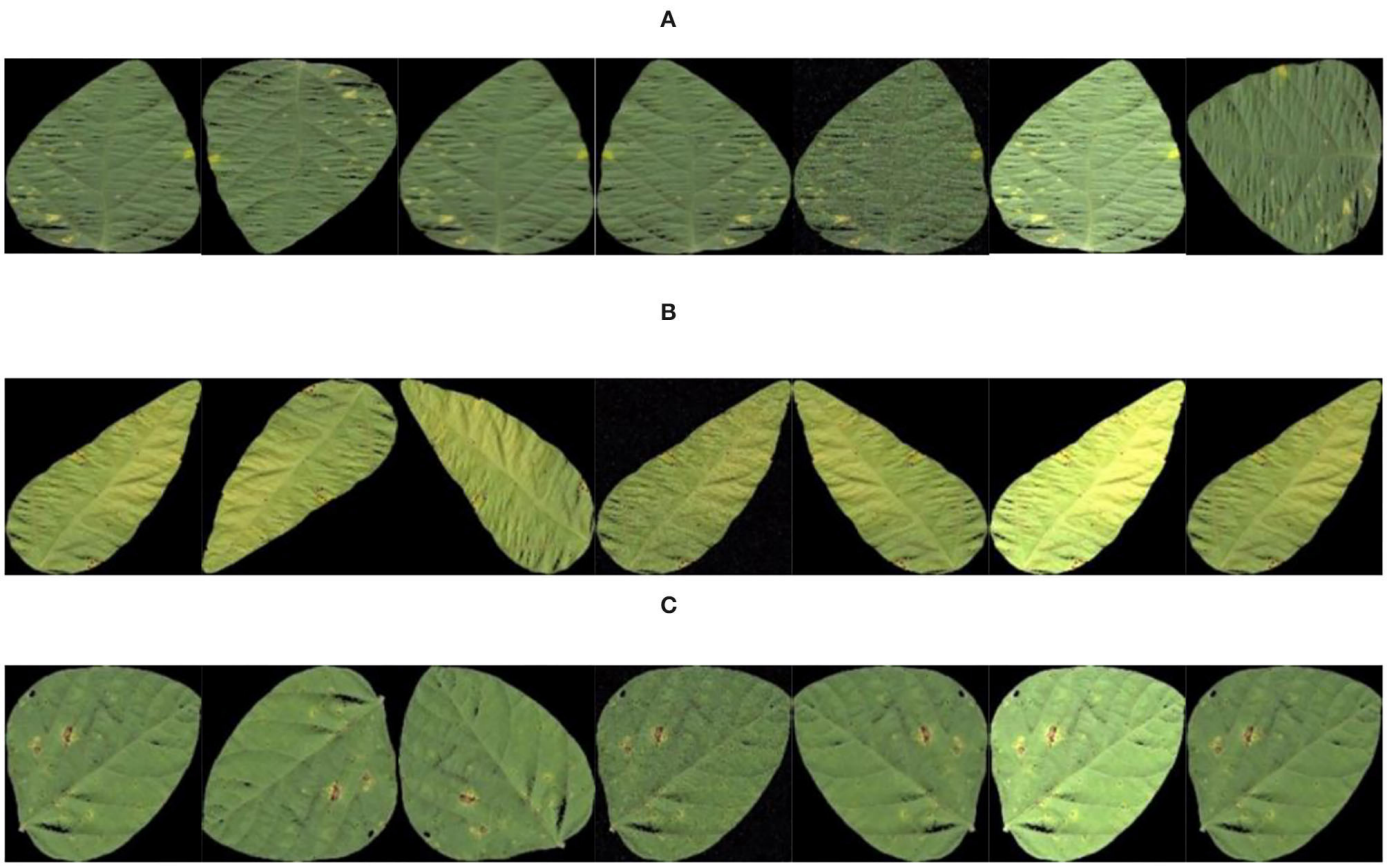
Data enhancement results. **(A)** Soybean brown leaf spot; **(B)** soybean frogeye leaf spot; **(C)** soybean phyllosticta leaf spot.

The image example of soybean disease single leaf after data enhancement is shown in Figure 9.

523 images of soybean leaf diseases were acquired in field. The threshold segmentation, region labeling, and a variety of data enhancement methods were used to expand the sample number based on the 523 images. Finally, a total of 39,446 image samples were obtained through sample expansion, which randomly divided into training set, validation set and test set, according to the ratio of 7:2:1. Among them, 27,616 were training sets, 7,887 were verification sets and 3,943 were test sets. Three kinds of soybean disease images were added with coding labeled by [1,0,0], [0,1,0], and [0,0,1]. The data sample size and division of various disease images are shown in Table 2.

**Table 2:** Sample coding and quantity division of various diseases.

**Disease categories**	**Coding labels**	**Training sets**	**Verification sets**	**Test sets**
Soybean brown leaf spot	[1,0,0]	9,072	2,592	1,296
Soybean frogeye leaf spot	[0,1,0]	8,351	2,385	1,192
Soybean phyllosticta leaf spot	[0,0,1]	10,193	2,910	1,455

## Construction of a Novel Residual Network Based on Attention Mechanism

### Residual Neural Network ResNet18

In recent years, convolutional neural networks have achieved good results in image classification and recognition. Compared with traditional classification methods, convolutional neural networks developed on AlexNet, VGG, GoogleNet, and ResNet achieve a higher recognition rate. However, deep neural networks have the problems of gradient disappearance and gradient explosion. In order to avoid the gradient problem caused by increase in network layers, identity mapping is introduced in a residual network.

**Figure 10:**
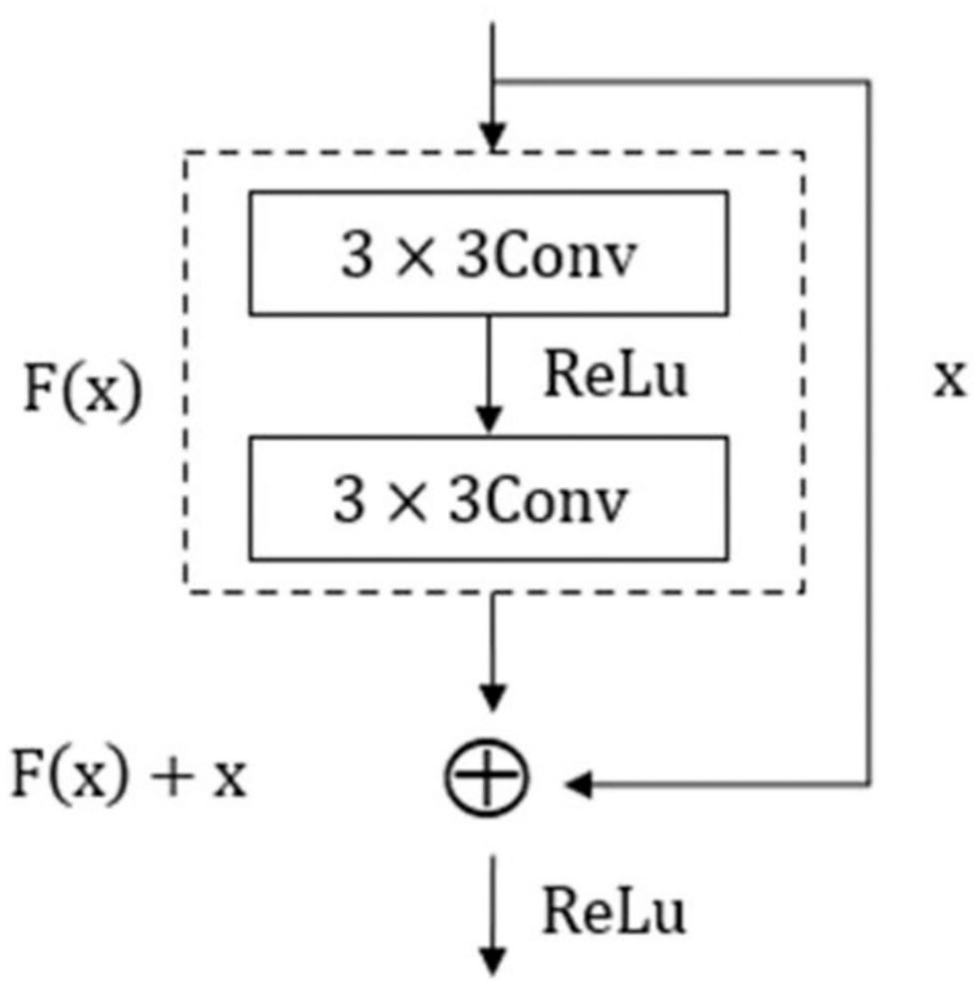
Residual structure.

ResNet18 is an excellent residual network model with low computational burden. The unit structure is shown in Figure 10. The input of its structure was x, and after learning *F*(*x*) through two residual errors consisting of 3 × 3 convolution layers and activation function Relu, the superposition of identity mapping x results in the actual output of *H*(*x*) = *F*(*x*)+*x*. The objective of residual structure optimization was *F*(*x*) = *H*(*x*) − *x*, and the ideal optimization was to approximate it to 0. This unit made up for the deficiency of information distortion and loss in the process of image information processing, which greatly improved the learning ability of the network. The residual network can effectively solve a series of problems caused by the gradient increase, such as gradient disappearance and gradient explosion, and it is essential to increase network depth if the accuracy need enhancing further in a training model, which will lead to increase in width and complexity of the network as well as expansion of memory capacity.

**Figure 11:**
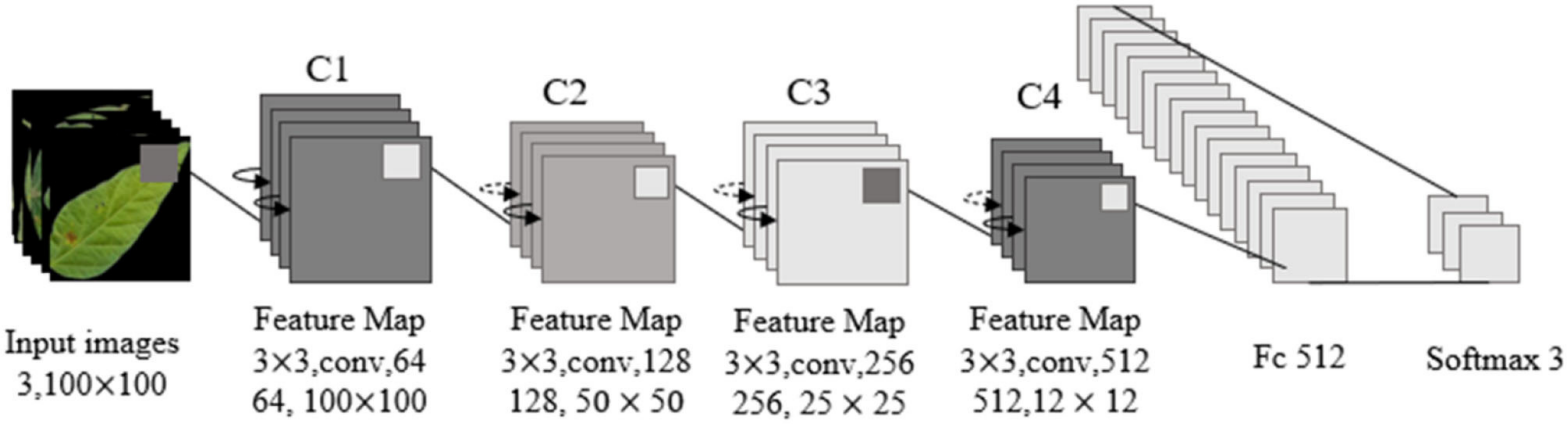
Resnet18 network structure.

The overall network structure of ResNet18 included an input layer, a residual module, a pooling layer, a full connection layer, and an output discrimination layer. A residual module was composed of an infrastructure layer of grouping residual connections. Each residual module included two groups of 3 × 3 convolution layers, batch normalization layers, and activation functions. ReLU was used as an activation function but not as an output discriminant layer. Softmax was adopted to recognize soybean leaf diseases. The structure of the network is shown in Figure 11.

In Figure 11, the size of image input by the ResNet18 network is 100 × 100 × 3. In view of the characteristics of small class spacing of leaf disease images, Sixty-four 3 × 3 convolution kernels were adopted to extract features, reduce parameters, and increase feature expression levels. Then, four 64-channel residual modules with a step size of 1 were used to convolve features, and the output characteristic graph Cl was obtained with a size of 100 × 100 × 64. Four 128-channel residual modules with a step size of 2 were used to convolve C1, and the output feature graph C2 was obtained with a size of 50 × 50 × 128. Four 256-channel residual modules with a step size of 2 were used to convolve C2, and the output feature graph C3 was obtained with a size of 25 × 25 × 256. Four residual modules with 512 channels and a step size of 2 were used to convolve C3, and the output feature graph C4 was obtained with a size of 12 × 12 × 512. After the global maximum pooling of C4, three neurons in the Softmax classification layer were obtained through 512 neurons in the whole connection layer, and the three neurons corresponded to the three kinds of soybean leaf diseases in this study.

### A Novel Residual Attention Network

**Figure 12:**
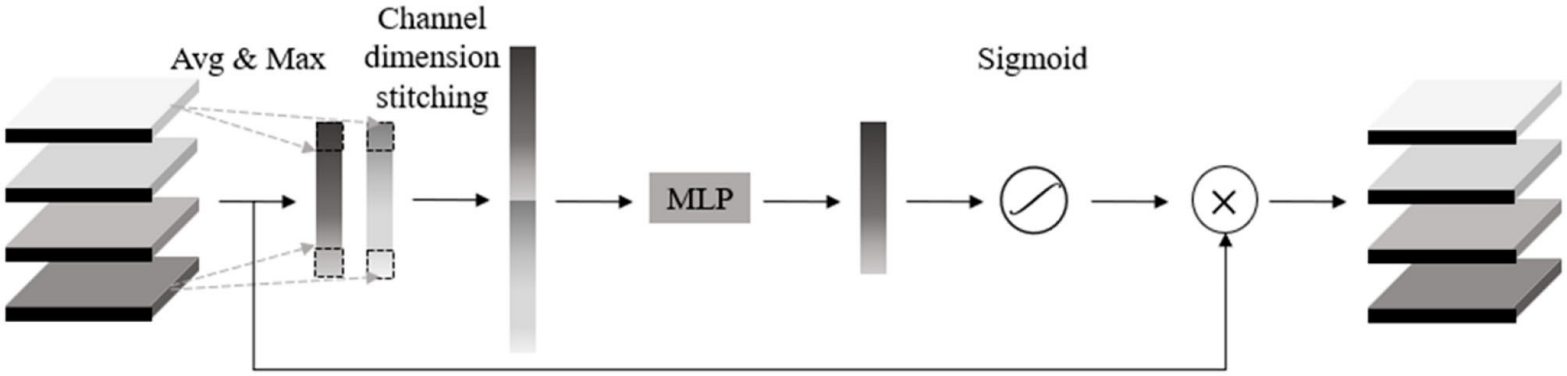
Channel attention module.

**Figure 13:**
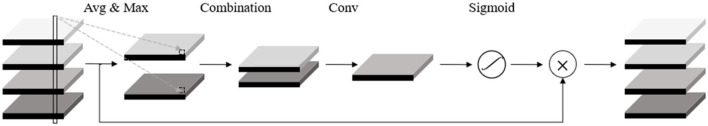
Spatial attention module.

A residual attention network (RANet) was proposed by combining the attention mechanism with ResNet18 to identify soybean leaf diseases. The attention mechanism was realized by constructing an attention module, which consisted of two parts: a parallel pooled channel attention module (Figure 12) and a parallel pooled spatial attention module (Figure 13). Parallel pooling referred to the simultaneous use of average pooling and max pooling, which described the information contained in feature channels more effectively. Average pooling compressed the spatial dimensions of the input feature map, aggregated the spatial information, and maximized pooling to collect unique object features, which can infer more detailed attention on the channel.

#### Attention Module

First of all, a channel attention module was used to operate global pooling for the input characteristic pattern with a size of h × w × c on the spatial dimension. After the pooling operation, the size of the characteristic pattern was 1 × 1 × c, and the characteristic value of the average pooling operation mainly described the background information of the image, and the pooling operation with the largest eigenvalue mainly described the texture information of the image. Using two kinds of pooling operations simultaneously, the information contained in the feature channel can be described more effectively. After joining the two groups of pooling outputs in channel dimension, MLP mapping was conducted to express the weights of importance for each channel, and then sigmoid function was adopted to constraint weight range to the interval between 0 and 1. Finally, the input feature was weighted to enhance the expression of diseased areas while inhibiting the expression of useless information to further improve the disease characteristic's contribution to the decision and recognition accuracy.

The output characteristic expression of the channel attention module is shown in Equation (29):


(29) $$\begin{array}{l} F^{\prime }\;=\;M_{\text{c}}\left(F\right)\;⊗\;F \end{array}$$

Where *F*′ represented the output features of the channel attention module, *F* represented the mechanism input features, *M*_c_(*F*) represented the channel attention features, and ⊗ represented the multiplication operation for the elements of the feature matrix. The expression of *M*_c_(*F*) was:


(30) $$\begin{array}{l} M_{\text{c}}\left(F\right)\;=\;\sigma \left(MLP\left(AvgPool\left(F\right)\right)\;+\;MLP\left(MaxPool\left(F\right)\right)\right)\\ \;=\;\sigma \left(W_{1}\left(W_{0}\left(F_{avg}^{c}\right)\right)\;+\;W_{1}\left(W_{0}\left(F_{\max}^{c}\right)\right)\right) \end{array}$$

where $F_{avg}^{c}$ represented the average pooling feature of *F*, $F_{\max}^{c}$ represented the maximum pooling feature of *F*, *W*_0_, and *W*_1_ represented the weight added to the pooling feature, and σ represented the sigmoid function.

The spatial attention module first conducted average pooling and maximum pooling operations on the channel dimension for the input characteristic pattern with a size of H × W × C. After the operation, the size of the two characteristic patterns was H × W × 1, which reduced the number of additional parameters and merged the two one-dimensional feature images into a two-dimensional characteristic pattern based on the channel. Then, the two-dimensional characteristic pattern was reduced to a one-dimensional channel characteristic using the 7 × 7 convolution. In addition, a mask map contained in the location information was extracted to be constrained by the sigmoid function for application in the input characteristic pattern to obtain the new characteristic pattern, which can inhibit the information expression of other areas and enhance the information expression of diseased areas.

The output characteristic expression of spatial attention module was:


(31) $$\begin{array}{l} F^{\prime \prime }\;=\;M_{\text{s}}\left(F^{\prime }\right)\;⊗\;F^{\prime } \end{array}$$

where, *F*″ represented the output feature of spatial attention module, *F*″ represented the input feature of spatial attention module, and $M_{\text{s}}\left(F^{\prime }\right)$ represented the spatial attention feature of *F*″. Where, the expression of $M_{\text{s}}\left(F^{\prime }\right)$ was:


(32) $$\begin{array}{l} M_{s}\left(F^{\prime }\right)\;=\;\sigma \left(f^{7\times 7}\left(\left[AvgPool\;\left(F^{\prime }\right);\;MaxPool\;\left(F^{\prime }\right)\right]\right)\right)\\ \;=\;\sigma \left(f^{7\times 7}\left({F^{\prime }}_{avg}^{s};{F^{\prime }}_{\max}^{s}\right)\right) \end{array}$$

where ${F^{\prime }}_{avg}^{s}$ represented the average pooling characteristic of *F*′, ${F^{\prime }}_{\max}^{s}$ represented the maximum pooling characteristic of *F*′, *f*^7×7^ represented the 7 × 7 convolution operation of ${F^{\prime }}_{avg}^{s}$ and ${F^{\prime }}_{\max}^{s}$, and σ represented the sigmoid function.

#### A New Residual Attention Network, RANet

**Figure 14:**
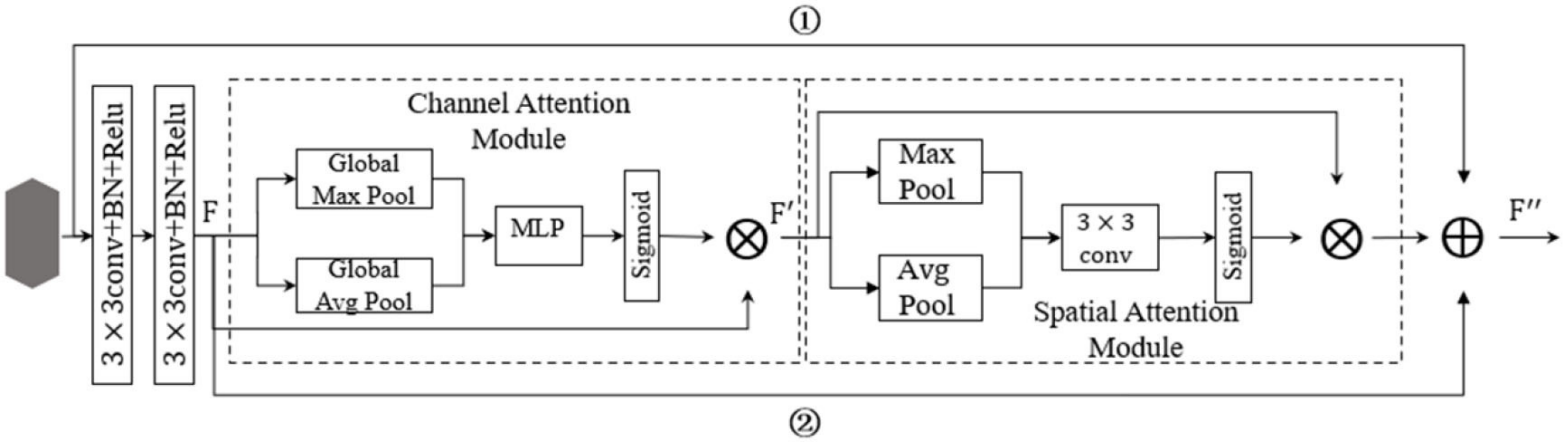
Residual attention layer (RAL) structure.

In this study, residual structure, channel attention, spatial attention mechanism, and shortcut connection were combined to construct a new residual attention layer (RAL), which is shown in Figure 14.

In Figure 14, image characteristics are extracted with two continuous convolutions to reduce the number of parameters added by the attention module. After the attention module was embedded in the two convolutions, the image characteristics extracted by two continuous convolution layers were taken as the input values of the attention module. Then, the attention module was applied to filter the input features using Equations (29)–(32). The optimized characteristics were obtained, that is, the effective weight was increased, and the invalid weight was reduced, so as to enhance the expression ability of disease regions. In Figure 14, the establishment of a new shortcut links step ① and step ②, can avoid subduction occurred on characteristic values when normalized by attention module. The whole RAL and attention module can be constructed as identity mapping to strengthen the network structure and performance degradation. The network can realize the adaptive combination of the attention module and convolution part by constructing identity mapping. In addition, the attention module will weigh the eigenvalues within a range of (0, 1), resulting in the reduction of the eigenvalues. The output *F*′ of the RAL was the sum of the input feature of the residual attention layer, the output *F* of the continuous convolution of the two layers, and the output characteristic values of the attention module.

**Figure 15:**
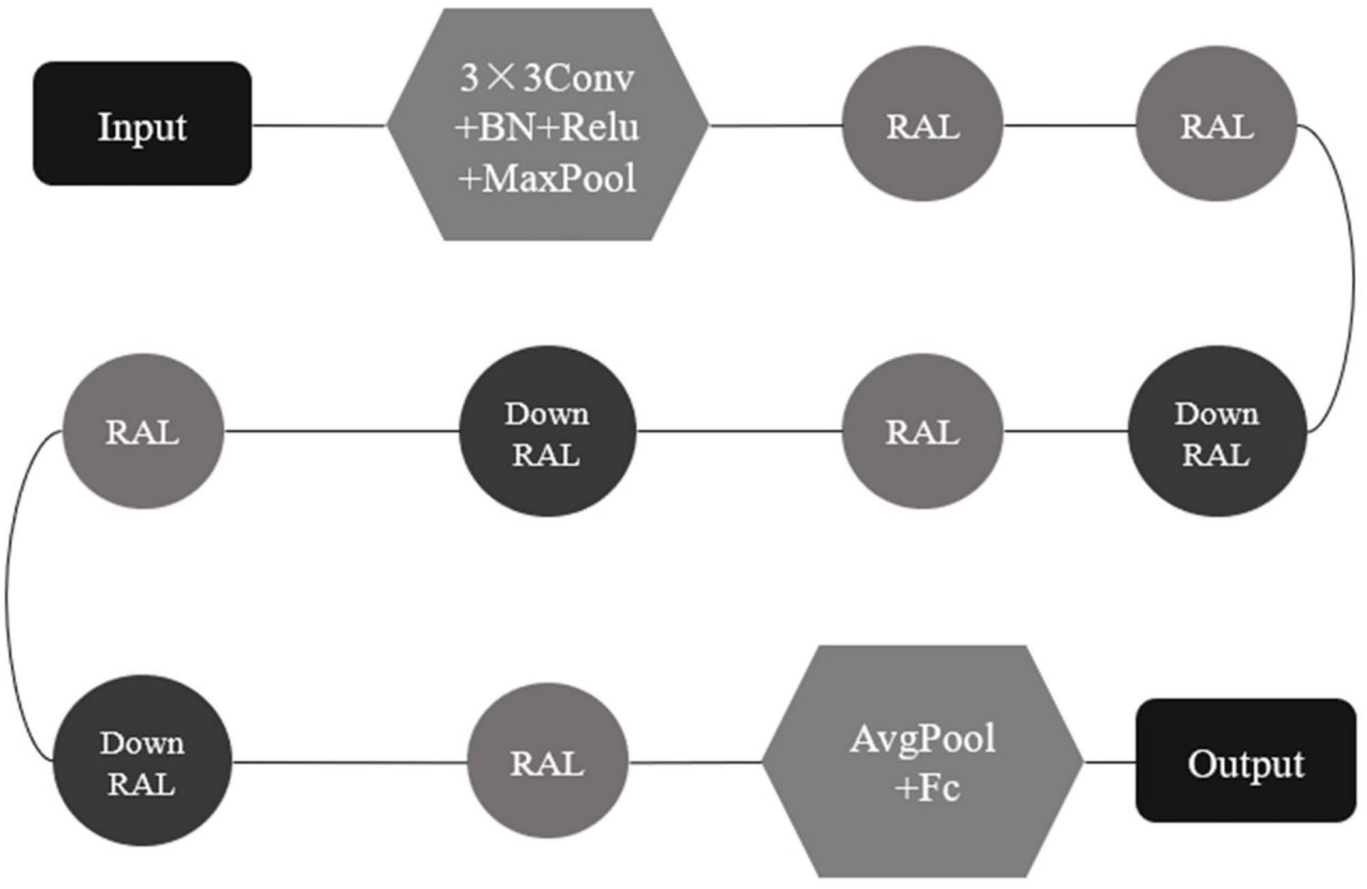
Residual attention network (RANet) structure.

According to the network structure of ResNet18, the mechanism of increasing attention and shortcut connection was embedded to form a RAL with more network constraints, and the RAL was embedded into ResNet18 to replace the residual module and build a novel residual attention network, RANet (Figure 15).

The whole structure of the RANet shown in Figure 15 includes an input layer, a network preprocessing layer, a RAL, a down RAL, a full connection layer, and an output discriminant layer. The down RAL represented that the step size of 3 × 3 convolution in the RAL module was 2 (The default step size of convolution in the RAL was 1), so that the characteristic matrix was reduced to half the size of the original matrix. The step size of the convolution kernel was set to 2 in the interlayer to compress the characteristic matrix instead of the pooling layer. The characteristic pattern was downsampled to ensure that the features extracted from the RAL were transformed into higher-level and more abstract features to reduce computation when decreasing the dimension of the characteristic vector.

### Optimizer

In this article, an adaptive motion estimation algorithm (Adam) optimizer was used to train network parameters. The Adam optimizer was designed based on independent adaptive learning rates for different parameters by calculating the first and second moment estimates of the gradient, which updated the variables using an exponential method to reduce the moving average of the gradient. Adam calculated the adaptive learning rate of each parameter, which stored the square of the past gradient and kept the moving mean of the past gradient index. Parametric decay factors β_1_ and β_2_ controlled the exponential decay rates of the moving mean values that were estimated using the first moment and second order original matrix of the gradient.

The specific steps of the Adam optimizer to train network parameters were as follows:

① To search and optimize each parameter, a moment vector *m*_*t*_ and an exponentially weighted infinite norm *v*_*t*_ must be maintained; *m*_*t*_ and *v*_*t*_ were initialized to 0 at the beginning of the search:


(33) $$\begin{array}{l} {\text{m}}_{0}\;=\;0,\;v_{0}\;=\;0 \end{array}$$

② We executed iteratively within the time t starting from *t* = 1. Each iteration computed a new set of parameter values x, from *x*_*t*−1_ to *x*_*t*_, to update all parameters by vector operation. Taking updating a parameter as an example, we first calculated the gradient (partial derivative) of the current time step:


(34) $$\begin{array}{l} g_{t}\;=\;f^{\prime }\left(x_{t\;-\;1}\right) \end{array}$$

(34) where *g*_*t*_was the gradient of the previous time step.

We updated the first moment with gradient *g*_*t*_ and hyperparameter β_1_:


(35) $$\begin{array}{l} m_{t}\;=\;\beta_{1}m_{t\;-\;1}\;+\;\left(1\;-\;\beta_{1}\right)g_{t} \end{array}$$

where $g_{t}^{2}$ was the square gradient, β_1_ was the decay factor of the first momentum.

We updated the second moment with square gradient $g_{t}^{2}$ and hyperparameter β_2_:


(36) $$\begin{array}{l} v_{t}\;=\;\beta_{2}v_{t\;-\;1}\;+\;\left(1\;-\;\beta_{2}\right)g_{t}^{2} \end{array}$$

where β_2_ was the decay factor of the infinite norm. β_1_ and β_2_ were used to control the first- and second-order momentum.

③ As *m*_*t*_ and *v*_*t*_ were initialized to 0 in step ①, *m*_*t*_/*v*_*t*_ will be biased to 0, especially in the initial training stage, so it was necessary to correct the deviation of gradient mean *m*_*t*_/*v*_*t*_ to reduce the influence of the deviation on the initial training stage.

The deviation correction of the first and second moments was carried out, taking the first moment as the starting point:


(37) $$\begin{array}{l} m_{t}^{\prime }\;=\;\frac{m_{t}}{1\;-\;\beta_{1}^{t}} \end{array}$$

Second moment:


(38) $$\begin{array}{l} v_{t}^{\prime }\;=\;\frac{v_{t}}{1\;-\;\beta_{2}^{t}} \end{array}$$

Where $\beta_{1}^{t}$ and $\beta_{2}^{t}$ decayed according to schedule during the iteration of the algorithm, and:


(39) $$\begin{array}{l} \beta_{1}^{t}\;=\;{\left(\beta_{1}\right)}^{t},\;\beta_{2}^{t}\;=\;{\left(\beta_{2}\right)}^{t} \end{array}$$

Finally, we iteratively calculated and updated parameters:


(40) $$\begin{array}{l} x_{t}\;=\;x_{t-1}\;-\;\alpha \frac{m_{t}^{\prime }}{\sqrt{v_{t}^{\prime }}\;+\;\varepsilon } \end{array}$$

Where α was the step size hyperparameter (learning rate), ε was a smoothing term, a small value to prevent the denominator from being 0 and ensure that no error was divided by zero.

The relevant hyperparameters of the experimental model in this study were set as follows: the learning rate of α was 0.001, which was adjusted by exponential attenuation. The attenuation factor β_1_ of the first momentum was 0.9, the attenuation factor β_2_ of the infinite norm was 0.99, and the smoothing term ε was 1E−8. Each batch of 64 images was used for training.

The cross entropy loss function was used to represent the difference between the real value and the predicted value. The cross entropy was used to evaluate the difference between the current training probability distribution and the real distribution. The smaller the distance between the actual output (probability) and the expected output (probability), the closer the two probability distributions.

The cross entropy loss equation was as follows:


(41) $$\begin{array}{l} C\;=\;-\frac{1}{n}\;\sum_{x}\left[y\ln\;a\;+\;\left(1-y\right)\ln\;\left(1\;-\;a\right)\right] \end{array}$$

(41) where C represented the cross entropy loss, y was the expected output, and α was the actual output of the neuron. α = σ(*z*) when z = ∑ω_*j*_ * *x*_*j*_ + *b*.

The cross entropy loss was used to differentiate the weights and bias terms:


(42) $$\begin{array}{l} \frac{\partial C}{\partial \omega_{j}}\;=\;\frac{1}{n}\;\sum_{x}x_{j}\left(\sigma \left(z\right)\;-\;y\right) \end{array}$$

(43) $$\begin{array}{l} \frac{\partial C}{\partial b}\;=\;\frac{1}{n}\;\sum_{x}\left(\sigma \left(z\right)\;-\;y\right) \end{array}$$

In the derivative of cross entropy loss to the weight ω_*j*_ and bias term *b* above, the update of weight was affected by error σ(*z*) − *y*. When the error was large, the update of weight was fast.

## Analysis of Experimental Results

### Soybean Disease Identification Process

**Figure 16:**
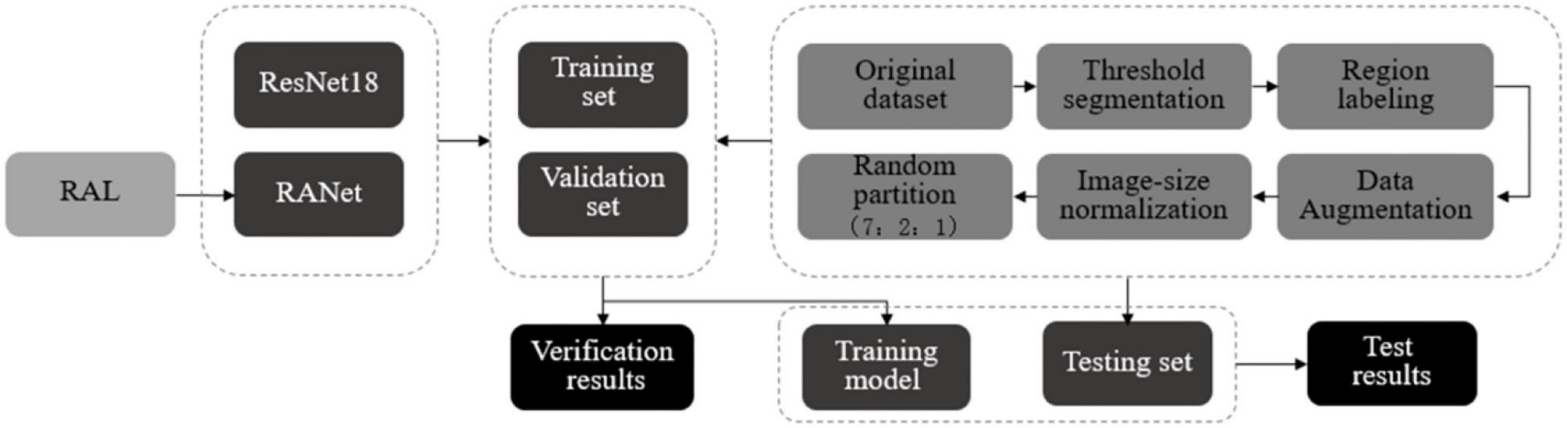
Disease identification process.

After threshold segmentation, region labeling, data enhancement, and size normalization, the images of soybean leaf diseases were divided into training set, validation set, and test set according to the ratio of 7:2:1 randomly. The training set generated and saved the training model on the network model. The accuracy and loss values of the training set and the validation set in the training process were important factors to evaluate model efficiency. The test set called the model generated by training, in which the recognition accuracy, accuracy, recall rate, and other indicators were obtained to quantitatively evaluate the performance of the model. The basic recognition process of each network on soybean disease image is shown in Figure 16.

### Experimental Environment

A RANet model was constructed based on ResNet18 and machine learning framework TensorFlow for recognition of soybean leaf diseases. In the experiment, the computer was configured with a 64-bit Windows 10 operating system and a 4-GB display card (NVIDIA GeForce GTX 1050 Ti). The software environment was Anaconda3 (64-bit), CUDEV10, Python 3.8, and Tensorflow-GPU 2.0.

### Training Process and Result Analysis

#### Training Set and Verification Set

There were 39,446 soybean disease images in this experiment. The soybean disease image set was randomly divided into training set + verification set and test set according to the ratio of 9:1. The training and verification sets participated in the training process together, but the two sample sets were randomly divided in strict accordance with the ratio of 7:2 during the training process. The soybean disease image set was divided according to the ratio of 9:1, the training set + verification set totalled to 35,503 and then randomly divided according to the ratio of 7:2. A total of 27,616 training sets were used to train models in the training process, including 9,072 for soybean brown leaf spot, 8,351 for soybean frogeye leaf spot, and 1.0193 for soybean phyllosticta leaf spot. A total of 7,887 validation sets were used to generalize the approximate the models during training, including 2,592 for soybean brown leaf spot, 2,385 for soybean frogeye leaf spot, and 2,910 for soybean phyllosticta leaf spot. The three kinds of soybean disease images were added coding labels in the training process by [1,0,0], [0,1,0], and [0,0,1], respectively.

#### Training Process and Result Analysis

**Figure 17:**
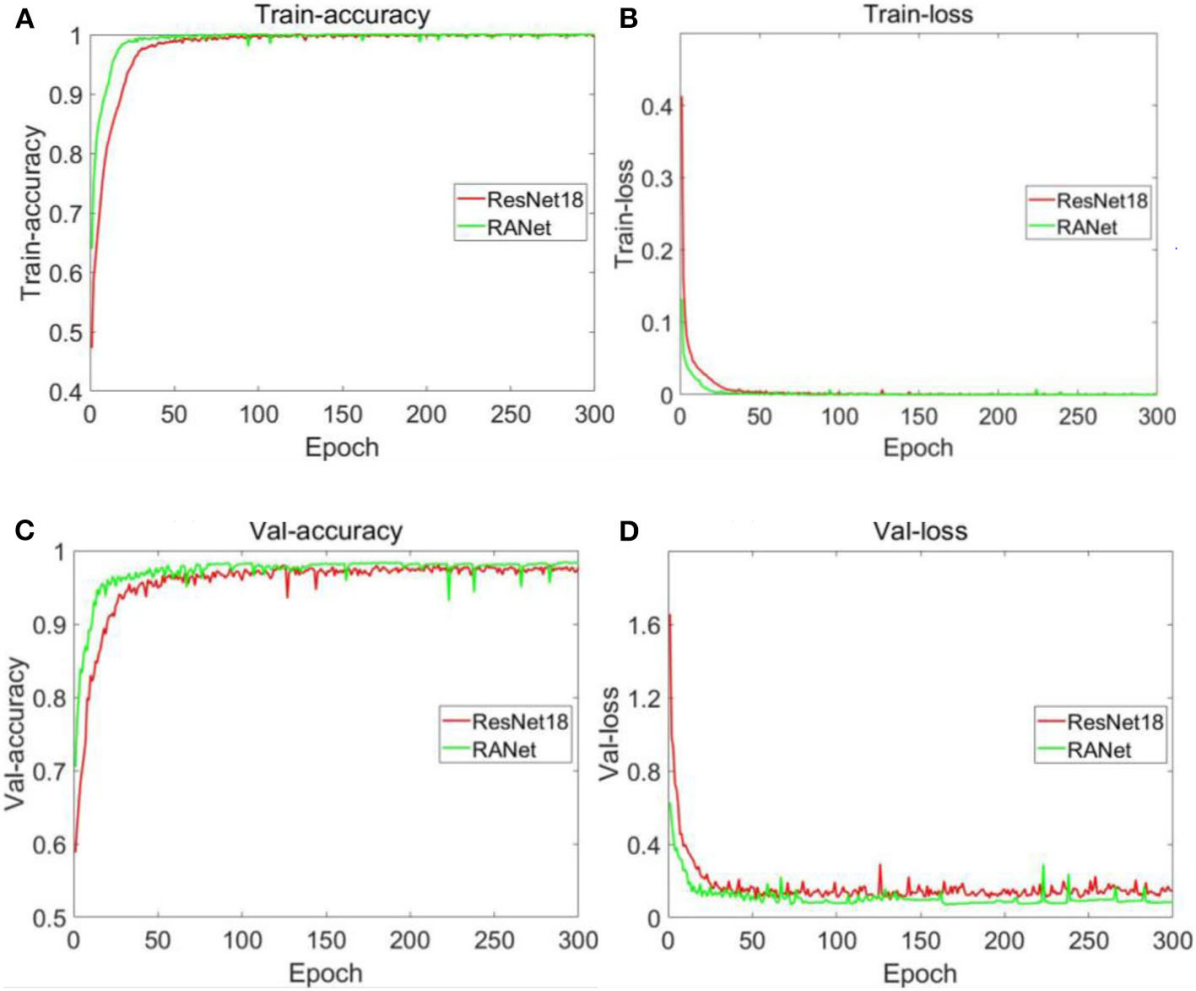
Changes in accuracy and loss values of training sets and validation sets of different models. **(A)** Accuracy of training set. **(B)** Loss value of training set. **(C)** Accuracy of validation set. **(D)** Loss value of validation set.

In order to speed up the training and improve the accuracy of the model, the input sample size of soybean disease of the two network models in this experiment was adjusted to 100 × 100 pixels according to the actual operating environment. During the training of the model, the batch processing (batch size) of the training samples was 64, and the training batch (epoch) was 300. The ReLu activation function was used to normalize the data of each batch by adding batch normalization. The Adam optimizer was selected for the model, and the learning rate was 0.001. The two network models used the same training and validation set sample size, same training batch, and same activation function. For the network structure of ResNet18 and RANet in the training process, the accuracy of the training set was 99.85 and 100%, respectively. The accuracy rate of the test set was 97.34 and 98.49%, respectively. The training set was used to train from scratch. Changes in accuracy and loss values of the algorithm training set and the validation set under different training methods are shown in Figure 17.

Figure 17A shows that the accuracy curve of ResNet18 varied from 0.472502 to 0.998451 with a range of 0.525949 and began to converge when the number of iterations was 31. The accuracy curve of RANet varied from 0.639518 to 1 with a range of 0.360482. When the number of iterations was 17, RANet started to converge. From the variation range of the accuracy of the two models on the training set and the number of iterations during convergence, RANet was 68.54% of the variation range of the accuracy and 54.84% of the convergence times of ResNet18, indicating that RANet had a good convergence effect and fast convergence rate in the training set. As shown in Figure 17B, the curve of ResNet18's loss value varies from 0.000466 to 0.412772 with a range of 0.412306. It began to converge when the number of iterations was 34. The curve of RANet's loss value varies from 1.37E^−06^ to 0.132611 with a range of 0.132610. The convergence started when the number of iterations was 19. From the variation range of the loss value of the two models in the training set and the number of iterations during convergence, RANet was 32.16% of the variation range of the accuracy and 55.88% of the convergence times of ResNet18, indicating that RANet had good convergence effect and fast convergence speed in the training set. As shown in Figure 17C, the accuracy curve of ResNet18 varies from 0.623433 to 0.976623 with a range of 0.35319 and begins to converge when the number of iterations is 44. The accuracy curve of RANet varied from 0.704816 to 0.983143 with a range of 0.278327. The convergence started when the number of iterations was 29. From the variation range of accuracy of the two models in the validation set and the number of iterations during convergence, RANet was 78.8% of the variation range of accuracy and 65.91% of the convergence times of ResNet18, indicating that RANet had a good convergence effect and a fast convergence rate in the validation set. As shown in Figure 17D, the loss value curve of ResNet18 varies from 0.145472 to 1.655084 and begins to converge when the number of iterations is 32, with a variation range of 1.509612. The loss value curve of RANet varied from 0.085767 to 0.626781, with a variation range of 0.541014. The convergence started when the number of iterations was 20. From the variation range of the loss value of the two models in the verification set and the number of iterations during convergence, RANet was 35.84% of the variation range of the accuracy and 62.5% of the convergence times of ResNet18, indicating that RANet had good loss convergence effect and fast convergence speed in the verification set. According to the above analysis in Figure 17, compared with ResNet18, RANet had better convergence effect of accuracy rate and loss value in the training and verification sets.

By comparative analysis of the accuracy of the training and validation sets under the two training methods shown in Figures 17A,C, RANet's curve convergence speed is significantly faster than ResNet18's. Compared with ResNet18, RANet with attention mechanism could effectively learn the characteristics of soybean diseased areas and improve the recognition accuracy of the algorithm more quickly. Characteristic learning ability and generalization ability were better. RANet had fast convergence speed, and the shortcut connection saved characteristic learning time. By comparing the changes in loss values of the training and verification sets under the two training methods shown in Figures 17B,D, the training method with the attention mechanism can accelerate the training and verification process. The above analysis results indicated that RANet had good training stability and high efficiency in the two network models in this experiment.

### Simulation Test and Result Analysis

#### Testing Set

In this experiment, 39,446 soybean disease image samples were randomly divided into 3,943 test sets according to the ratio of 9:1 to evaluate the performance of the model, including 1,296 for soybean brown leaf spot, 1,192 for soybean frogeye leaf spot, and 1,455 for soybean phyllosticta leaf spot. The soybean disease samples in the test set had no intersection with the training and validation sets.

#### Simulation Test and Result Analysis

The sample size of soybean disease images in the test set was also adjusted to 100 × 100 pixels, and the accuracy of the network structure of ResNet18 and RANet in the test set was 97.34 and 98.49%, respectively, in the training process. The accuracy of the two network models in the test sets of three soybean diseases is shown in Table 3.

**Table 3:** Accuracy of various disease test sets.

**Disease categories**	**Accuracy/%**	
	**ResNet18**	**RANet**
Soybean brown leaf spot	96.86	98.15
Soybean frogeye leaf spot	98.56	98.91
Soybean phyllosticta leaf spot	96.61	98.42
Average value	97.34	98.49

Table 3 shows that the identification accuracy of the three kinds of diseases of the RANet model was 0.35 and 1.81% higher than that of the ResNet18 model, and the average identification accuracy of the RANet model was 1.15% higher than that of the ResNet18 model. It was because the RANet model extracted the features of input information. In addition, the importance of characteristics was distinguished, so that the network can reduce the learning of regions unrelated to distinguishing diseases, and more attention (network resources) can be applied to the disease features; thus, disease characteristics can be extracted more carefully and recognition effect can be improved. The RANet model in the experiment was structured as channel attention module + spatial attention module. The mask map used to enhance constraints on input feature images in the spatial attention module was constructed based on all feature images, and the input can be adjusted according to the information contained in the characteristic images. Therefore, the characteristic image input into the spatial attention module was constrained at the channel level according to the information contained in the characteristic images. Therefore, in the construction of the mask map, characteristic maps containing disease information contributed more to the mask map, so that the mask map can better describe the spatial location of disease features and then apply more network resources to the disease areas to improve the ability of the convolution part to extract disease characteristics.

**Figure 18:**
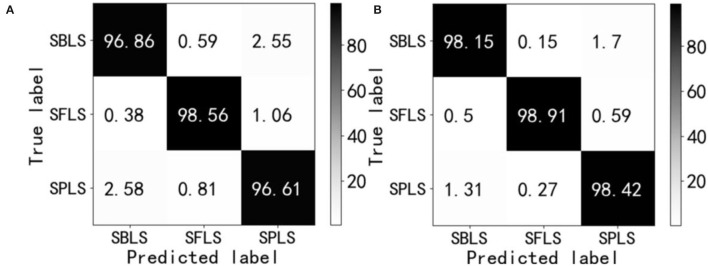
Confusion matrix. Soybean brown leaf spot (SBLS); soybean frogeye leaf spot (SFLS); Soybean phyllosticta leaf spot (SPLS). **(A)** ResNet18. **(B)** RANet.

Further comparison was made between the two network models visualized by the confusion matrix in the test set, and the results were statistically analyzed. The statistical results are shown in Figures 18A,B. In the field of machine learning, an obfuscation matrix is a case analysis table summarizing the predicted results of classification models, and a specific matrix is used to show the visual effect of the performance of supervised learning algorithms. The column represents the actual category of the sample, the row represents the predicted category of the sample, and each value in Figure 18 is the probability of predicting the column category into the row category. The value of the diagonal is the probability of correct prediction.

The results of the confusion matrix in Figure 18 show that there are 6 kinds of misrecognition in each of the three disease test sets. The recognition error rate and value of ResNet18 and RANet were 7.97 and 4.43%, respectively, and the recognition error rate of RANet was 3.54% lower than that of the ResNet18 model. The misidentification of the two models mainly occurred in the classification of soybean brown leaf spot and soybean frogeye leaf spot. Some characteristics of soybean brown leaf spot and soybean frogeye leaf spot were similar, so it was necessary to classify the characteristics more carefully or use fewer features to realize identification, which increased the difficulty of identification.

**Figure 19:**
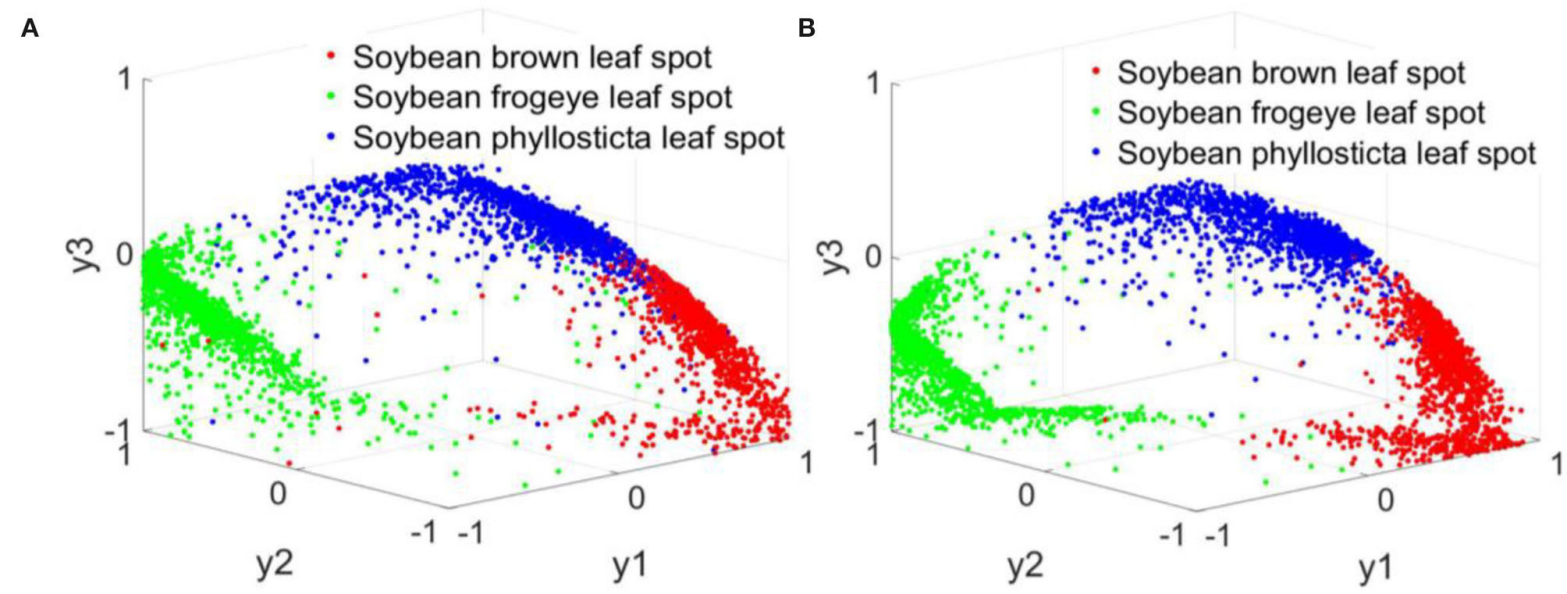
Normalization results of simulation calculation. **(A)** ResNet18. **(B)** RANet.

During the simulation experiment, the actual output value of ResNet18 and RANet network corresponded to the decoding mapping rule of the sole heat code of soybean disease type: when the network output was *y*_1_ = *y*_max_(*y*_1_, *y*_2_, *y*_3_), the corresponding soybean disease type code was [1,0,0], indicating soybean brown leaf spot; when the network output was *y*_2_ = *y*_max_(*y*_1_, *y*_2_, *y*_3_), the corresponding soybean disease type code was [0,1,0], indicating soybean frogeye leaf spot; when the network output was *y*_3_ = *y*_max_(*y*_1_, *y*_2_, *y*_3_), the corresponding soybean disease type code was [0,0,1], indicating soybean phyllosticta leaf spot. By comparing the maximum value of the output of the network to the favorable segment, the output value was established to the code vector, and the soybean disease category was resolved to establish a model method for fast recognition of soybean disease. Figure 19 shows the calculation results of the established ResNet18 and RANet models.

The three colors in Figure 19 represent the three soybean diseases. The actual output values of nodes in three columns of the ResNet18 and RANet models showed that only one soybean disease in each column has a maximum network output value.

Table 4 shows that the two network models are similar in size. Compared with the ResNet18 model, RANet, with the addition of the attention mechanism, saved 20.56% of the average recognition time of each disease image in the test set, although the training time was sacrificed.

**Table 4:** Comparison of training and recognition performance of different models.

**Model**	**Training time/h**	**Model size/MB**	**Mean recognition time/s**
ResNet18	9.05	42.75	0.0647
RANet	15.23	42.75	0.0514

Since precision and recall were a pair of contradictory measures with their own limitations, it was not reasonable to use either of them alone to evaluate the merits of the model. Only by comprehensively considering both can the model be objectively evaluated. F1 was the weighted harmonic average of precision and recall. Therefore, F1 value was introduced as a comprehensive evaluation index to balance the effects of precision and recall and evaluate a classifier comprehensively.

Precision was the percentage of samples that were predicted to be positive:


(44) $$\begin{array}{l} \text{Precision }=\;\frac{\text{TP}}{\text{TP }+\text{ FP}} \end{array}$$

where TP represented the positive sample predicted by the model as a positive class, FP represented the negative sample predicted by the model as a positive class, and the recall represented the percentage of positive samples that were predicted to be positive:


(45) $$\begin{array}{l} \text{Recall }=\;\frac{\text{TP}}{\text{TP }+\text{ FN}} \end{array}$$

where FN represented the positive sample predicted by the model as a negative class.

F1-values were taken into account for both precision and recall:


(46) $$\begin{array}{l} \text{F}1\;=\;\frac{2\;\times \text{ Precision }\times \text{ Recall}}{\text{Precision }+\text{ Recall}} \end{array}$$

The closer the values of precision, recall and F1 to 1, the better the performance of the model.

The precision, recall, and F1-values of ResNet18 and RANet were calculated according to the confusion matrix and Equations (44)–(46). The performance evaluation value of each model is shown in Table 5.

**Table 5:** Model performance evaluation.

**Model**	**Precision**	**Recall**	**F1**
ResNet18	97.34	97.34	97.35
RANet	98.50	98.49	98.52

Table 5 lists the comparison results of the two different models. Compared with ResNet18, RANet with the attention mechanism improved the precision by 1.16, recall by 1.15, and the value of F1 by 1.17, which showed that the three performance evaluation indexes of RANet were higher than those of ResNet18. The performance of the network model was improved under the combination of attention mechanism, shortcut connection, and convolutional neural network.

## Discussion

In this study, a fast method of recognition of soybean disease based on an improved residual network was proposed. The accuracy of the simulation experiment reached 98.49%, and a good effect was achieved in the fast recognition of soybean leaf diseases. The specific analysis of the experimental results was as follows:

1. Data pre-processing. Due to the large proportion of background in the disease images in this experiment, in order to reduce the adverse impact of background features on image recognition (classification) results in the process of characteristic extraction, the OTSU method was adopted to effectively eliminate the interference information generated by the background. A regional calibration algorithm was used to get the leaf disease images, which highlighted the disease areas effectively. Data enhancement methods including rotation, mirror imaging, and adding noise as well as filtering were applied to expand the experimental samples, which provided sufficient and reliable experimental data sets for the new convolution neural network.
2. Structure of residual attention layer. After the continuous convolution layer of the residual structure in ResNet18, a novel method for constructing a RAL was proposed by adding the attention mechanism and the shortcut connection. This method can enhance the expression of effective information in disease area images, avoiding reduction of the eigenvalue when the attention module normalized the eigenvalue and further improving the problem of network performance degradation.
3. Establishment of recognition model. The ResNet18 deep learning model was used to establish a novel residual attention network model for soybean disease recognition. The model took the pre-processed disease images as the input variable of the input layer node. The output layer adopted the form of independent thermal coding and used the strong adaptive learning characteristic of the neural network to construct a fast recognition model of soybean leaf diseases. The scale of the input layer and the number of nodes of the output layer were determined according to the different sizes of images extracted from different research objects and different recognition targets. Although there were some differences in the structure and scale of the constructed models, the Adam algorithm was used to obtain the structural parameters of the deep learning model and reconstruct an accurate and reliable residual attention network model.
4. Error analysis of the model. The model realized the recognition of 3 kinds of soybean leaf diseases, but the simulation results showed 3 errors, the main reasons included: characteristics of the diseases exhibited similarities and redundancy, which increased the difficulty of disease recognition, so more detailed area characteristics were needed to realize accurate and efficient recognition. The data pre-processing method still needs improvement to avoid errors in soybean disease recognition model due to color distortion and geometric distortion caused by machine vision system and shooting angle. On the premise of avoiding model overfitting, optimizing the learning algorithm of a new residual attention network is one of the efficient ways to improve the recognition accuracy of the model.
5. Application and promotion. The simple structure and strong adaptability of the model are conducive to its practical application and promotion. For different plants and disease species, special models of specific disease species and plants and special recognition models can be reconstructed using strong automatic learning characteristics. Thus, based on the research content of this study, increasing plant species and diseases is the first work in our future research. Then, sequence analysis using the method of amplifying ITS by PCR will be studied to expand the scope of plant disease recognition combining the adaptability of a deep learning model with prior knowledge of plant protection experts. In addition, plant disease recognition technology will be improved to achieve the applicability of recognition models. Finally, the constructed recognition model will be integrated into a portable mobile terminal for fast recognition of soybean leaf diseases.

## Conclusion

In this study, three kinds of soybean leaf diseases were used as experimental data sets, and the iterative method, OTSU method, and local threshold method were analyzed to segment the performance indexes of original images of the soybean leaf diseases. The OTSU method was selected to remove image background, and the region labeling method was used to obtain the single leaf area image of soybean leaf diseases. On the basis of image sample expansion and sample division with the digital image enhancement method, a new model of residual attention network for soybean disease recognition was established by adding attention mechanism and shortcut connection based on a residual neural network, ResNet18.

(1) The OTSU method was used to extract disease images effectively, reducing the influence of background in the disease images. Effective image samples were obtained by labeling the area of a leaf image highlighting the effective information of disease areas. Rotation, mirror imaging, adding noise, and filtering were conducted to expand the image samples, and the effective samples increased to 39,446 pieces, which not only increased sample size but also enriched the type of training samples and further provided sufficient and reliable experimental data sets for the new convolutional neural network to extract image characteristic information.

(2) After the continuous convolution layer of the residual neural network (ResNet18), a RAL was constructed by adding the attention mechanism and the shortcut connection. The RAL was inserted into ResNet18 to replace the residual structure, and a new residual attention network model (RANet) was established. ResNet18 and RANet were used to verify and test the performance of the model in the test set. The average recognition rate of RANet was 98.49%, the F1-value was 98.52, and the recognition time was 0.0514 s. Compared with the ResNet18, the recognition rate was increased by 1.15%, the F1-value was increased by 1.17, and the disease image recognition time saved was 0.0133 s. Thus, the proposed model was fast, accurate and stable enough to recognize soybean leaf diseases.

## Data Availability Statement

The original contributions presented in the study are included in the article/supplementary material, further inquiries can be directed to the corresponding author/s.

## Author Contributions

HG: conceptualization. HG, XM, and MY: methodology. MY: validation and writing (original draft preparation). HG, XM, MY, ML, and TZ: formal analysis. HG and XM: resources. HG and MY: writing (review and editing). ML and TZ: visualization. All authors have read and agreed to the published version of the manuscript.

## Funding

This research was funded by the National Natural Science Foundation of China (Grant No: 31601220), Natural Science Foundation of Heilongjiang Province, China (Grant Nos: LH2021C062 and LH2020C080), and Heilongjiang Bayi Agricultural University Support Program for San Heng San Zong, China (Grant Nos: TDJH202101 and ZRCQC202006).

## Conflict of Interest

The authors declare that the research was conducted in the absence of any commercial or financial relationships that could be construed as a potential conflict of interest.

## Publisher's Note

All claims expressed in this article are solely those of the authors and do not necessarily represent those of their affiliated organizations, or those of the publisher, the editors and the reviewers. Any product that may be evaluated in this article, or claim that may be made by its manufacturer, is not guaranteed or endorsed by the publisher.

## References

[B1] Cen H. Y. Zhu Y. M. Sun D. W. Zhai L. Wan L. Ma Z. H. (2020). Current status and future perspective of the application of deep learning in plant phenotype research. Trans. Chin. Soc. Agric. Eng. (Trans. CSAE). 36, 1–16. 10.11975/j.issn.1002-6819.2020.09.001

[B2] Chang K. F. Hwang S. F. Ahmed H. U. (2018). Disease reaction to Rhizoctonia solani and yield losses in soybean. Can. J. Plant Sci. 98, 115–124. 10.1139/cjps-2017-0053

[B3] Gao Q. J. Li C. B. Jin X. Li Y. Y. Wu H. P. (2021). Research on the traceability system of tea quality and safety based on blockchain. J. Anhui Agric. Univ. 48, 668–673. 10.13610/j.cnki.1672-352x.20210824.002

[B4] Guo D. Zhang H. Yang J. T. Yu L. Y. Wu B. J. Li M. M.. (2021). Occurrence status and green control counter measure for diseases and insect pests of soybeans in Shandong Province. Soybean Sci. Technol. 4, 27–30. 10.3969/j.issn.1674-3547.2021.04.006

[B5] Han B. Zeng S. W. (2021). Plant leaf image recognition based on multi-feature integration and convolutional neural network. Comput. Sci. 48, 113–117. 10.11896/jsjkx.201100119

[B6] He X. Li S. Q. Liu B. (2021). Identification of grape leaf diseases based on multi-scale residual neural network. Comput. Eng. 47, 285–291. 10.19678/j.issn.1000-3428.0057818

[B7] Kannojia S. P. Jaiswal G. (2018). Effects of varying resolution on performance of CNN based image classification an experimental study. Int. J. Comp. Sci. Eng. 6, 451–456. 10.26438/ijcse/v6i9.451456

[B8] Lei X. Pan H. Huang X. (2019). A dilated CNN model for image classification. IEEE Access. 99, 1. 10.1109/ACCESS.2019.2927169

[B9] Li L. P. Shi F. P. Tian W. B. Chen L. (2021). Wild plant image recognition method based on residual network and transfer learning. Radio Eng. 51, 857–863. 10.3969/j.issn.1003-3106.2021.09.003

[B10] Liang Y. Li B. B. Jiao B. (2019). Research and implementation of CNN Based on TensorFlow. IOP Conf. Ser. Mater. Sci. Eng. 490, 558–564. 10.1088/1757-899X/490/4/042022

[B11] Ma Y. Shan Y. G. Yuan J. (2021). Tomato leaf disease recognition based on three-channel attention network. Sci. Technol. Eng. 21, 10789–10795. 10.3969/j.issn.1671-1815.2021.25.031

[B12] Shang Y. H. Yu Y. J. Wu G. (2021). Plant diseases recognition based on mixed attention mechanism. J. Tarim Univ. 33, 94–103. 10.3969/j.issn.1009-0568.2021.02.013

[B13] Singh A. Ganapathysubramanian B. Singh A. K. (2016). Machine learning for high-throughput stress phenotyping in plants. Trends Plant Sci. 21, 110–124. 10.1016/j.tplants.2015.10.015 26651918

[B14] Sun Y. Y. Zhao L. M. Zhang W. Zhang C. B. (2021). Research progression utilization of soybean heterosis. Soybean Sci. Technol. 6, 26–35. 10.3969/j.issn.1674-3547.2021.06.006

[B15] Wang C. S. Zhou J. Wu H. R. Teng G. F. Zhao C. J. Li J. X. (2020). Identification of vegetable leaf diseases based on improved Multi-scale ResNet. Trans. Chin. Soc. Agric. Eng. (Trans. CSAE). 36, 209–217. 10.11975/j.issn.1002-6819.2020.20.025

[B16] Wang X. Y. Zhang C. Zhang L. (2021). Recognition of similar rose based on convolution neural network. J. Anhui Agric. Univ. 48, 504–510. 10.13610/j.cnki.1672-352x.20210628.001

[B17] Xu Y. Li X. Z. Wu Z. H. Gao Z. Liu L. (2021). Potato leaf disease recognition *via* residual attention network. J. Shandong Univ. Sci. Technol. (Natl. Sci). 40, 76–83. 10.16452/j.cnki.sdkjzk.2021.02.009

[B18] Zhang S. Huai Y. J. (2016). Leaf image recognition based on layered convolutions neural network deep learning. J. Beijing Forest. Univ. 38, 108–115. 10.13332/j.1000-1522.20160035

